# Bayesian Model Averaging with Change Points to Assess the Impact of Vaccination and Public Health Interventions

**DOI:** 10.1097/EDE.0000000000000719

**Published:** 2017-09-28

**Authors:** Esra Kürüm, Joshua L. Warren, Cynthia Schuck-Paim, Roger Lustig, Joseph A. Lewnard, Rodrigo Fuentes, Christian A. W. Bruhn, Robert J. Taylor, Lone Simonsen, Daniel M. Weinberger

**Affiliations:** From the a Department of Statistics, University of California, Riverside, CA; b Department of Biostatistics, Yale School of Public Health, New Haven, CT; c Sage Analytica, Bethesda, MD; d Department of Epidemiology of Microbial Diseases, Yale School of Public Health, New Haven, CT; e Dpto. de Epidemiología, DIPLAS, Ministerio de Salud, Chile; and f Department of Global Health, Milken Institute School of Public Health, George Washington University, Washington, DC.

## Abstract

**Background:**

Pneumococcal conjugate vaccines (PCVs) prevent invasive pneumococcal disease and pneumonia. However, some low-and middle-income countries have yet to introduce PCV into their immunization programs due, in part, to lack of certainty about the potential impact. Assessing PCV benefits is challenging because specific data on pneumococcal disease are often lacking, and it can be difficult to separate the effects of factors other than the vaccine that could also affect pneumococcal disease rates.

**Methods:**

We assess PCV impact by combining Bayesian model averaging with change-point models to estimate the timing and magnitude of vaccine-associated changes, while controlling for seasonality and other covariates. We applied our approach to monthly time series of age-stratified hospitalizations related to pneumococcal infection in children younger 5 years of age in the United States, Brazil, and Chile.

**Results:**

Our method accurately detected changes in data in which we knew true and noteworthy changes occurred, i.e., in simulated data and for invasive pneumococcal disease. Moreover, 24 months after the vaccine introduction, we detected reductions of 14%, 9%, and 9% in the United States, Brazil, and Chile, respectively, in all-cause pneumonia (ACP) hospitalizations for age group 0 to <1 years of age.

**Conclusions:**

Our approach provides a flexible and sensitive method to detect changes in disease incidence that occur after the introduction of a vaccine or other intervention, while avoiding biases that exist in current approaches to time-trend analyses.

## Abstract 2

Supplemental Digital Content is available in the text.

Pneumococcus *(Streptococcus pneumoniae*) causes an array of diseases, including pneumonia and invasive pneumococcal disease (IPD), resulting in almost 1 million childhood deaths annually in the pre-vaccine years.^1^ The first pneumococcal conjugate vaccine (PCV7), which targeted seven of the 90+ pneumococcal serotypes, was introduced in 2000 in the United States. Newer versions of the vaccine on the market, PCV10 and PCV13, target 10 and 13 serotypes, respectively, are now in widespread use worldwide. Accurate determination of PCV impact is necessary to support public health decision-making. In particular, many low- and middle-income countries are considering introducing or have already introduced PCVs. These countries will need information about the overall impact of PCVs as they decide whether to implement or continue to support PCVs in the future.

The ability to detect a vaccine-associated change in disease rates depends on the magnitude of the decline and the amount of unexplained variability in the data—a small change in noisy data is difficult to see. Analysis of bacterial surveillance data,^2,3^ as well as trends in national mortality,^4^ and hospitalization data^5^ have shown that the use of PCVs led to substantial declines in rates of IPD among both vaccinated children and unvaccinated older children and adults. Estimating the impact of PCVs on IPD is relatively straightforward because the decline is large and corroborated by laboratory testing. However, measuring the impact of PCVs against pneumonia is more difficult because the etiologic agent is rarely identified or recorded. Randomized controlled trials (RCTs) suggest that PCVs should have a relatively modest effect of 0.1%–10.8% against syndromic pneumonia hospitalizations among children <2 years of age.^1^ In contrast, some time-trend studies have reported declines of up to 40% in all-cause pneumonia hospitalizations.^6,7^ However, in observational studies of this type, changes in healthcare systems and socioeconomic improvements are all nonvaccine factors that may influence reported reductions, as could the specificity of the pneumonia case definitions. Robust methods that quantify the timing and magnitude of any changes that occur after vaccine introduction would help to strengthen estimates of vaccine impact and improve comparability between studies.

A common approach used to estimate vaccine impact is interrupted time series analysis, which involves using a regression model to evaluate changes in incidences or trends between selected pre- and post-vaccine years. This approach, however, requires two potentially problematic assumptions. First, it prespecifies the point in time when a change in the outcome is expected (typically at or shortly after vaccine introduction). This is problematic because it is uncertain when a vaccine will begin to exert a detectable effect, and the estimated reductions can depend greatly on the choice of the cut-off point. Also, if an unrelated trend begins shortly before vaccine introduction, this change could be incorrectly attributed to the vaccine. And a short-term spike in incidences that occurs shortly before vaccine introduction—such as the 2009 pandemic occurring just before PCV10 introduction in Brazil and PCV13 in the United States in 2010—could influence the estimated pre-vaccine trends and bias the results. These sources of bias are usually not well investigated. Second, investigators typically choose one model over all others, when in fact alternative models might describe the data equally well but nonetheless may yield different impact estimates.^8^ For example, a model that assumes an immediate, sudden decline in incidences due to vaccine might give biased results if, in fact, the decline is delayed or gradual. In practice, investigators do not know the structure of changes in incidence or which nonvaccine forces may be operating. Therefore, assuming that one model can describe the data adequately is not optimal.

To address these issues, we have combined change-point analysis^9^—a method to detect the timing and magnitude of changes in time series data—with Bayesian model averaging—a method to systematically integrate results from different model structures and covariates. Change-point modeling allows the data to reveal if and when any substantial change in the time series occurred, with fewer presuppositions. Bayesian model averaging obviates the need to choose a single “best” model by providing a weighted average of results from models fitted using different forms and combinations of covariates; it also provides insight into the importance of each covariate included in the change-point models. This approach allows assessment of the uncertainties in timing and magnitude of any changes found in the time series. Here, we demonstrate the usefulness—and limitations—of this approach by applying it to the assessment of PCV impact in the United States, Brazil, and Chile. We also provide the results of a Monte Carlo simulation study that assessed the accuracy and precision of our approach.

## METHODS

### Data Sources

We obtained monthly hospitalization data from three countries: the United States, Brazil, and Chile. We focused our study on children <5 years of age (stratified by <12, 12–23, and 24–59 months of age) because pediatric age groups are most likely to be directly affected by vaccination. PCV7 was introduced in the United States in February 2000, and PCV10 was introduced in Brazil and Chile in March 2010 and January 2011, respectively. See eAppendix (http://links.lww.com/EDE/B237) for details on data sources.

In the US data, we used any mention of the relevant International classification of diseases, ninth revision (ICD9) codes (eTable 1; http://links.lww.com/EDE/B237) in the hospitalization discharge records to define patients who had IPD and pneumococcal (lobar) pneumonia; for all-cause pneumonia, we used two definitions: the ICD9 definition that Griffin et al^10^ (2013) developed, and a less stringent but commonly used “any mention” definition (referred here as the “standard definition”). In Brazil and Chile, codes specifically indicating pneumococcal pneumonia and pneumococcal infection were rarely used, and secondary codes were not recorded; thus, we could only analyze ACP defined by the “standard” definition (eTable 1; http://links.lww.com/EDE/B237). To highlight the strengths and pitfalls of our approach, we also present results of the model fit to rotaviral enteritis and urinary tract infections (UTIs) for which no PCV benefits are expected. The analyses of these de-identified data were deemed exempt from review by the Human Investigation Committee at Yale School of Medicine.

### Bayesian Model Averaging with Change Points: Overview

We combined change-point models with Bayesian model averaging. We fit two types of change-point models^11–13^: one capturing sudden declines (change in mean) and the other capturing gradual declines over time (change in slope). To allow for the possibility that no significant change occurred, we also fit models with no change point. To capture time trends not explained by the change points, all models included a smooth function of time, which was estimated using a nonparametric mixed model approach, and therefore lead to inclusion of random effects (for details, see eAppendix; http://links.lww.com/EDE/B237). We also allow a set of covariates to be included in the model, and the outcome 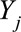 is assumed to follow a Poisson distribution with mean 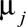.

The three model structures were as follows:


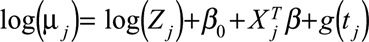
(No change point)(1)


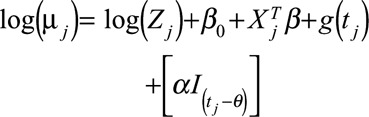
(Change in mean)(2)


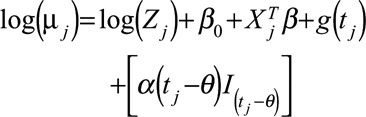
(Change in slope)(3)

where for time 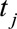, 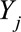
is the number of cases, 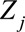 is an offset term representing the total number of hospitalizations excluding 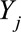, 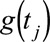 is a smooth function of time, 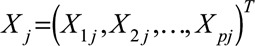 and 
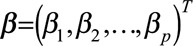
 are the vectors of covariates and *p* regression coefficients, respectively, 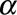 is the coefficient of change point, i.e., the change in expected counts due to the intervention that occurs at time 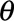, and 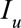 is an indicator function that is 1 when its subscript is greater than 0 and is 0 otherwise, and 
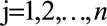
 with n as the number of time points.

For Bayesian model averaging, we fit each of the three model structures with all possible combinations of covariates and each candidate change point. This resulted in a large set of candidate models. For details of the estimation procedure, see eAppendix (http://links.lww.com/EDE/B237).

We placed two restrictions on our models. First, to minimize edge effects, which are typical in the analyses of time series data and can lead to biased results, we assumed the probability that a change point occurred in the first or last 6 months of the time series was 0. Second, in data applications where more than one change point was needed to capture the variation in the data, we required each point be separated by at least 12 months from all others, to avoid capturing short-term epidemic patterns.

### Estimating the Counterfactual Predictions

To estimate the decline in incidences that occurred after vaccine introduction, it is necessary to compare the model-averaged fitted incidences with an estimate of the counterfactual incidence (counterfactual prediction)—what would have been expected to occur if the vaccine was not introduced. We estimated the counterfactual incidence by using the model that was fit to the entire data.

While estimating the counterfactual predictions, there are three possible scenarios to consider: (1) no change in incidence; (2) change in incidence before the vaccine; and (3) change in incidence after the vaccine. If there were no changes or a change occurred before the vaccine, no change should be attributable to the vaccine, and the counterfactual predictions should align with model-averaged fitted values. In contrast, if the change occurred after vaccine introduction, this change should be attributed to the vaccine. To implement this idea in our models, we multiplied the regression coefficients of the change points (*α* in equations 2 and 3) by the sum of the posterior probabilities from the models that indicated a change occurred before the vaccine or no change at all. If the models with a change point before the vaccine fit the data better, we multiply *α* (equations 2 and 3) with a number close to 1 and the counterfactual predictions will be close to model-averaged fitted values. On the contrary, if the models with a change point after the vaccine fit the data better, we multiply *α* with a number close to 0, and the counterfactual predictions will be further away from model-averaged fitted values.

We obtained the incidence rate ratio (IRR)—a measure of the magnitude of the change for each time point—by dividing model-averaged fitted values by counterfactual predictions. We obtained 95% bootstrap confidence intervals for the IRR (for details, see eAppendix; http://links.lww.com/EDE/B237).

We assessed the uncertainty associated with the existence and location of each change point using the distribution of posterior probabilities. The sum of the posterior probabilities for the models with a change point gives an indication of the confidence that there was a substantial change in the time series data—posteriors close to 1 indicates strong evidence of a change, values close to 0 indicate strong evidence of no change, and values close to 0.5 indicate uncertainty about whether there was a change.

### Bayesian Model Averaging with Change Points: Applications

#### National-level Hospitalizations

We applied Bayesian model averaging with change points to the national-level hospitalization data from the United States, Chile, and Brazil. All analyses were stratified by age group, and separate models were fit for each disease outcome (eTable 1; http://links.lww.com/EDE/B237). In each analysis, the total number of hospitalizations in the relevant age group and year, excluding the hospitalizations for the outcome of interest, was used as the denominator. As a starting point for all of the analyses, we allowed a single change point at an unknown time. Because the resulting posterior model probabilities suggested that we needed two change points, i.e., we had a bimodal posterior distribution, to explain the patterns in the United States and Chile, we reanalyzed these data allowing up to two change points that were separated by at least 12 months. In Brazil, a health program (Pact for Health) targeting diarrhea and pneumonia began in 2006, and a 2008 healthcare delivery system reform affected the specificity of coding; thus, for Brazil, we included two fixed change points in January 2006 and 2008 (a dummy variable for before or after that time point) and allowed a single change point any time after January 2009. In the analysis of pneumonia and invasive pneumococcal disease, all models included harmonic terms with 6- and 12-month periods to capture the seasonal structure of the data. In addition, we included age-adjusted numbers of influenza hospitalizations as a covariate to control for the severity of different influenza seasons; numbers of influenza cases were aggregated over all age groups to provide an estimate of influenza activity across the population and avoid known age-specific coding biases.

#### Simulation Studies

We generated five sets of simulated time series that resembled observed time series in terms of number of monthly cases, seasonality, and degree of random unexplained variability but on which we imposed changes of known timing and magnitude. For each set, we generated 100 time series that followed a Poisson distribution, and parameters for this distribution were extracted from invasive pneumococcal disease and pneumonia time series from the United States, Chile, and Brazil using a Poisson regression model in PROC MCMC (SAS Inc., Cary, NC).^14^ The last simulation study used parameters obtained from Brazil pneumonia series and was used to demonstrate the performance of Bayesian model averaging with change points in the absence of a vaccine effect. For details on construction of simulated data, see eAppendix (http://links.lww.com/EDE/B237).

We evaluated the performance of the proposed models first in terms of IRR estimations by comparing the median estimated IRR of all 100 simulated data sets to the true IRR value at 12 months after the change point. At the same time point, we also assessed the uncertainty associated with IRR estimation through coverage of the 2.5 and 97.5 percentiles of estimated IRR values based on 100 simulation runs. We assessed the precision of our change-point estimates by comparing the true change-point locations to mean change-point locations, which were calculated as the average of the time points in each simulation run that had the largest posterior probability.

We compared the performance of Bayesian model averaging with change points in estimating IRR to interrupted time series approach in simulated data and real data applications. See eAppendix (http://links.lww.com/EDE/B237) for details.

Bayesian model averaging with change points was performed using the GAMM4 package in R V3.1.3 (R, Vienna, Austria),^15^ and a sample R code (http://links.lww.com/EDE/B238) along with a data set (http://links.lww.com/EDE/B239) is available as supplementary materials.

## RESULTS

### Performance of Models on Simulated Time Series

We applied Bayesian model averaging with change points to five sets of 100 simulated time series, which had different effect sizes and number of years observed (eTable 2; http://links.lww.com/EDE/B237). The method accurately detected the timing and magnitude of changes when vaccine effect was large (similar to the ~60% reduction seen in invasive pneumococcal disease in the United States^3^) (eFigure 1; http://links.lww.com/EDE/B237). The model accurately captured both the timing of the change and the magnitude of the decline after the simulated vaccine introduction. In the simulated data where there was no imposed vaccine effect, Bayesian model averaging with change points—correctly—did not detect a change in 69% of the simulated time series. The method performed well when estimating the change-point location(s) in simulated data where the imposed decline was smaller and several years of data before and after vaccine were available (the data were similar to ACP [definition in ^10^] in the United States and Brazil). However, when only 2 years of post-vaccine data were available, as was the case with simulated data that resembled all-cause pneumonia in Chile, IRR estimates were slightly biased. Finally, when the expected IRR was small, change-point estimate was less accurate, with up to a year between this estimate and true change point.

The comparison of Bayesian model averaging with change points with interrupted time series analysis (eTable 3; http://links.lww.com/EDE/B237) showed that for data with a single change point (similar to characteristics of Brazil all-cause pneumonia data), these methods gave mostly comparable results. With two change points, the results of interrupted time series analysis and Bayesian model averaging with change points were closest when the cut-off for interrupted time series was close to the mean second change point calculated with Bayesian model averaging with change points (eTable 2; http://links.lww.com/EDE/B237) (for details, see eAppendix; http://links.lww.com/EDE/B237).

### Estimates of Changes Following the Introduction of PCVs

**FIGURE 1.**
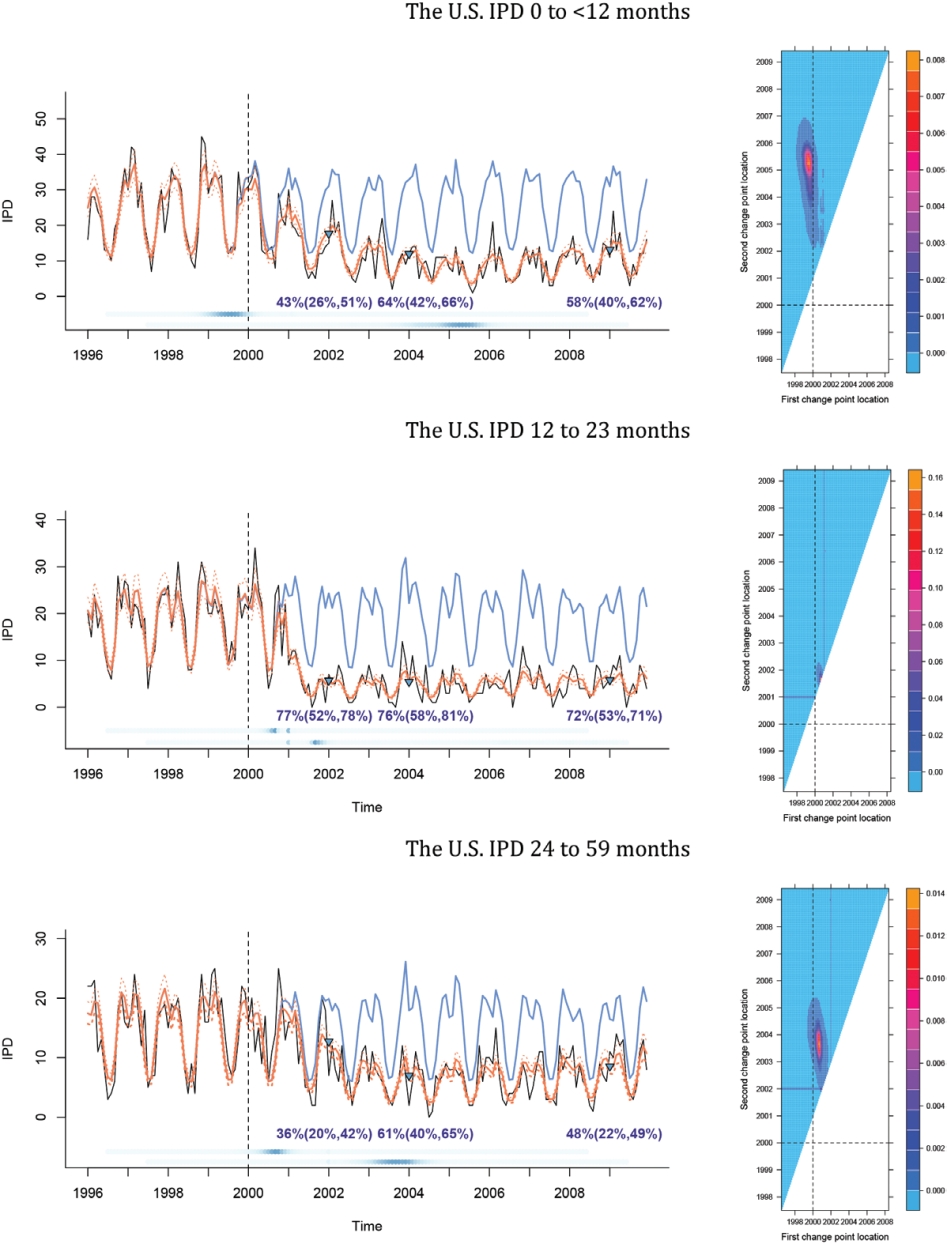
The plots on the left represent, IPD hospitalizations versus time for 10 US states by age group, showing observed IPD hospitalizations per month (black), model-averaged fitted values (orange, solid) with their 95% approximate pointwise confidence intervals (orange, dotted), and counterfactual predicted values (blue). The estimated decline at specific time points (green triangles) is shown, with their respective 95% bootstrap confidence intervals. The blue dots at the bottom represent the probability of a change occurring at that point. The color gets darker as the probability increases. The first and second sets of dots are for the first and second change points, respectively. The level plots on the right are posterior probabilities corresponding to the plots on the left for the locations of the first (*x* axis) and second (*y* axis) change points. The dashed lines represent the time that the PCV7 (January 2000) is introduced.

Within 24 months of vaccine introduction, large declines (36%–43%) had occurred in rates of invasive pneumococcal disease in the United States among children under 5 years of age. For invasive pneumococcal disease, the probability that at least one change point occurred after vaccine introduction was strong (probability of 0.999 for age groups <12-, 12–23-, and 24–59-month-olds). The posterior probabilities indicated that a second change point occurred 4–7 years after vaccine introduction as disease rates leveled out (Figure 1 and Table). By 24 months after PCV7 introduction, pneumococcal (lobar) pneumonia declined by 36%, 43%, and 32% among children <12, 12–23, and 24–59 months of age in the United States, respectively. The corresponding probabilities that a change occurred after PCV7 introduction were 0.977, 0.999, and 0.999 (eFigure 2; http://links.lww.com/EDE/B237; Table).

**TABLE.**
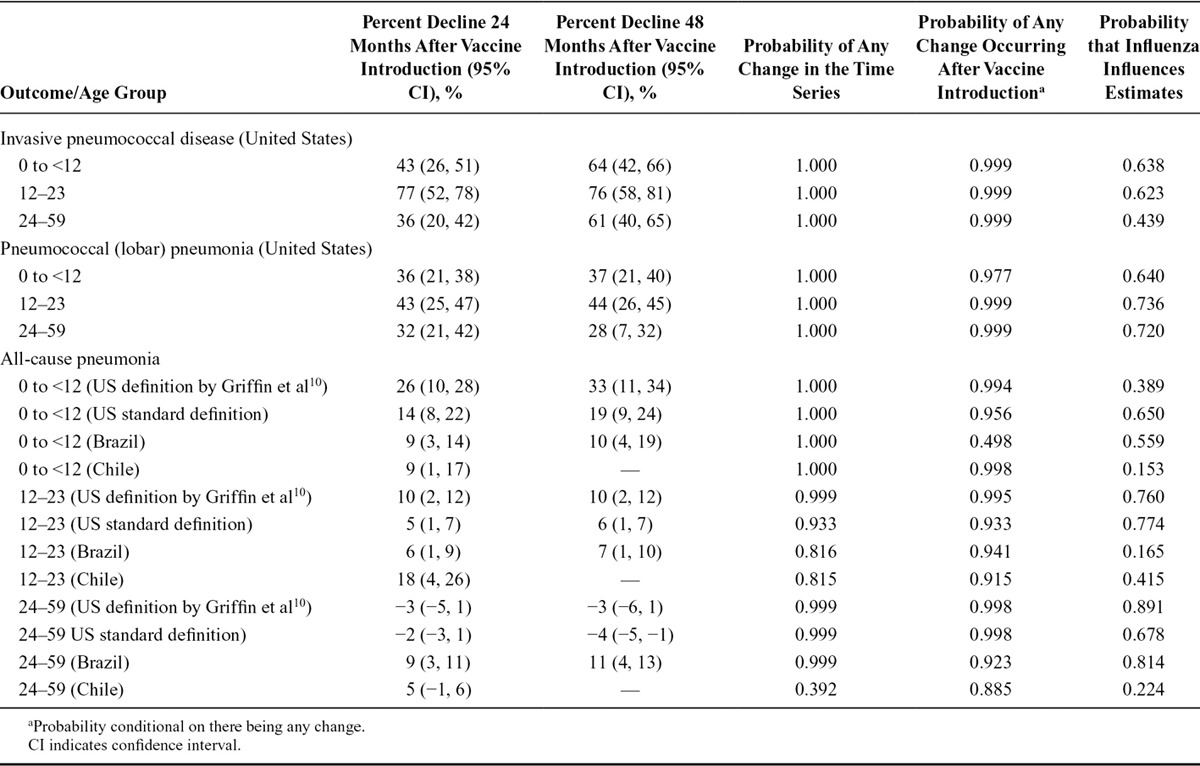
Estimated Percent Decline ([1-IRR] × 100) and Probabilities that Changes Occurred After Vaccination by Age Group, Country, and Outcome

**FIGURE 2.**
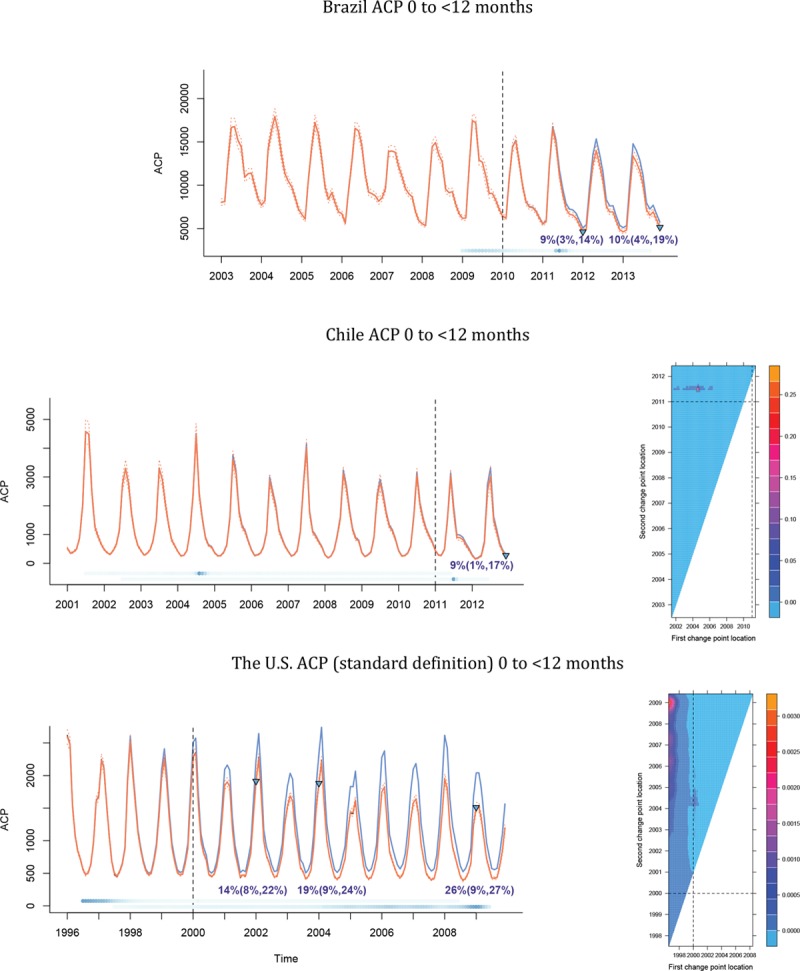
The plots on the right are ACP hospitalizations versus time for Brazil (first row), 10 US states (second row), and Chile (third row) for age group 0–12 months, showing observed ACP hospitalizations per month (black), model-averaged fitted values (orange, solid) with their 95% approximate pointwise confidence intervals (orange, dotted), and counterfactual predicted values (blue). The estimated decline at specific time points (green triangles) is shown, with their respective 95% bootstrap confidence intervals. The blue dots at the bottom represent the probability of a change occurring at that point. The color gets darker as the probability increases. In the second and third rows of the plot, the first and second sets of dots are for the first and second change points, respectively. The level plots on the right are posterior probabilities (in the second and third rows) corresponding to the plots on the left (in the second and third rows) for the locations of the first (*x* axis) and second (*y* axis) change points. The dashed lines represent the time that the pneumococcal conjugate vaccine is introduced.

All-cause pneumonia declined by 14%, 9%, and 9% among <12-month-olds in the United States, Brazil, and Chile, respectively, at 24 months after PCV introduction. The probabilities that a decline occurred after PCV introduction were 0.956, 0.498, and 0.998 in the United States, Brazil, and Chile, respectively. For older children, the probability of a decline after PCV introduction was in all three countries greater than 0.8. Using a definition of ACP that is more specific for pneumococcus,^10^ the decline in the Unites States was, as expected, larger at 26% (Figure 2; eFigures 3–6; http://links.lww.com/EDE/B237; Table).

Our method can make use of covariates to control for changes that are unrelated to vaccination. For example, for the stringent all-cause pneumonia definition^10^ in the United States, there was weak evidence that influenza (eTable 1; http://links.lww.com/EDE/B237) should be included in the model for <12-month-olds (probability = 0.389), while there was strong evidence (probability = 0.891) that it should be included in models for 24–59-month-olds (Table).

We compared the results of Bayesian model averaging with change points to an interrupted time series model (eTable 4; http://links.lww.com/EDE/B237). The estimates for the decline in pneumonia hospitalizations after PCV introduction obtained from the former method were generally closer to zero (i.e., smaller effects) (for details, see eAppendix; http://links.lww.com/EDE/B237).

### Estimates of Changes in Nonpneumococcal Outcomes

In the United States, Bayesian model averaging with change points did not detect a decline in rotaviral enteritis immediately after PCV introduction (as expected) but detected a decline in <12-month-olds after 2006, coincident with the introduction of the rotavirus vaccine. We also detected a decline in the number of UTI cases in the United States after 2000 among infants <12 months of age and among children 12–23 months of age. In Brazil, we did not detect any changes in UTI cases in infants <12 months of age after PCV introduction (eFigure 7 and eTable 5; http://links.lww.com/EDE/B237). These patterns highlight the need for caution when attributing trends from these types of analyses to a specific intervention.

## DISCUSSION

The benefits of a public health intervention are typically assessed from time series data using a regression model that compares disease rates and trends before and after the intervention. The advantage of Bayesian model averaging with change points over this traditional approach is two-fold. First, it estimates the time at which a change in incidence occurred rather than imposing a preselected time frame on the analysis, making it less likely that an unrelated trend from before vaccine introduction would be attributed to the vaccine. Second, it removes the need to choose a single “best” model structure to describe the data. In practice, analysts might not know the structure of change(s) in incidences or the covariates that describe the data best. We avoid this limitation by fitting a number of possible models with different combinations of covariates.

We have demonstrated the advantages of Bayesian model averaging with change points by assessing PCV impact in three different epidemiologically distinct settings. Two of these were middle-income countries for which we only had data on all-cause pneumonia, the pneumococcal outcome that is least specific and therefore most challenging to assess. We believe that this method could be used effectively to evaluate the impact of other vaccines and public health interventions in many such settings, including low-income countries in which the data are even more challenging.

Comparison of Bayesian model averaging with changes points and interrupted time series analysis indicates that both methods yield similar results when there is a single change point in the data and the latter correctly specifies this point as the cut-off. In practice, however, the exact point to interrupt the time series may not be optimal. Our results show that if the data are not interrupted at the correct time point, the estimate of interrupted time series will be biased. Moreover, it tends to estimate larger percent declines for data with more than one change point (eTable 3; http://links.lww.com/EDE/B237). For analysts who decided to perform interrupted time series as the primary analysis and where there is strong prior information about when the change should occur, we would suggest that Bayesian model averaging with change points would provide a useful secondary analysis with an additional confirmatory information (the timing of the change).

Because contact patterns between age groups are not uniform, and the etiology of pneumonia varies by age, the change-point locations and magnitudes could differ by age group. Therefore, we performed a separate analysis for each age group and disease outcome. Our analysis supported this approach as the declines in some disease outcomes are earlier in vaccinated children compared with unvaccinated adults (who are indirectly protected).

The estimated decline in invasive pneumococcal disease in children under 24 months of age was consistent with results from active surveillance studies; pneumococcal (lobar) pneumonia in the United States, an outcome that is more specific for pneumococcal pneumonia than all-cause pneumonia, declined in a similar pattern after PCV introduction. However, our estimates of the decline in all-cause pneumonia (standard definition) among children <24 months of age were notably smaller than some published estimates from trend studies. For example, Afonso et al^7^ (2013) estimated a 24%–29% decline in Brazil, and Grijalva et al^6^ (2007) found a 39% decline in all-cause pneumonia (stringent definition) in the Unites States. Some of the differences between our results and published studies could be due to differences in the definition of pneumonia used; in fact, we detected a larger decline (26%) when using the stringent all-cause pneumonia definition,^10^ suggesting that this definition is more specific to pneumococcus than the standard definition. Our estimates, however, are more consistent with the declines in clinical pneumonia estimated in RCTs.^1^ Importantly, when there is uncertainty about whether a trend begins before or after vaccine, Bayesian model averaging with change points is inherently more conservative in estimating the amount of change that occurred after vaccine than interrupted time series or other similar methods. Comparisons with these other methods can be helpful to highlight situations where the simpler interrupted time series approaches might be biased and further analytical attention is needed.

Covariates can be incorporated into Bayesian model averaging with change points to control for changes in incidence unrelated to vaccination. As an example, we included age-aggregated influenza hospitalizations (ICD10 J09–J11) as a covariate to control for the highly variable severity of seasonal influenza because a severe influenza season could bias IRR and change-point estimates for pneumonia (and potentially hospitalizations caused by pneumococcus specifically as well).^16,17^ For instance, a severe flu season immediately after vaccine would lead to an increase in pneumonia hospitalizations and mask the effect of the vaccine, while a severe season before vaccine would exaggerate the effect. The influenza covariate allows us to subtract out the effect of influenza from the estimates. Although including this covariate might bias the estimates if the vaccine reduces hospitalizations coded as influenza or if coding patterns changed after vaccine, it is still important to control for influenza season severity for the reasons mentioned above. By summing the weights of models that include influenza, we obtained an estimate of the probability that influenza appreciably influenced the results; values close to 1 were strong evidence that it did, values near 0 were strong evidence that it did not, and values near 0.5 were inconclusive (Table).

Our approach has limitations. First, Bayesian model averaging with changes points requires an investigator to determine the number of change points before fitting the models; we are developing a more flexible approach that will estimate the appropriate number of change points. Second, its algorithm is computationally heavy due to the smooth functions of time used in the models. Therefore, fully Bayesian approaches that employ spike-and-slab priors will be explored to overcome this burden. Third, more simulation studies are required to determine the effects of sample size, unexplained variability, and effect size on the accuracy of the estimates. Finally, an inherent limitation of both Bayesian model averaging with change points and interrupted time series is the need to assume that any trends that occurred before vaccine would continue indefinitely into the post-vaccine period. Incorporating information on contemporaneous control variables into the analysis^18^ could help to relax these assumptions and generate more credible counterfactual estimates.

As with any trend analysis, the results of Bayesian model averaging with change points should be interpreted cautiously. Although it strengthens inferences on vaccine impact by estimating the timing and magnitude of the change after the intervention, unrelated factors can still influence trends in incidences. For instance, we found changes in UTI hospitalizations in the United States (but not in Brazil) after PCV introduction that cannot plausibly be attributed to PCV. One solution to this problem would be to identify an appropriate set of comparison outcomes that share the same set of confounders as the disease of interest but is not influenced by the intervention.^19^ Identifying these outcomes is challenging in practice, as using an inappropriate outcome might lead to a misleading association between the vaccine and the comparator outcome. We chose UTI and rotaviral enteritis to demonstrate the challenge in selecting such outcomes and to reiterate that caution should be exercised when attributing any change found in pneumonia-related hospitalizations to the vaccine.

In conclusion, we have proposed a flexible approach to evaluate the impact of PCVs, as well as any public health intervention. Our approach estimates the location and magnitude of the change in disease incidence by fitting a number of alternative models that account for different potential covariates and different structures of the change. Unlike current approaches, our method removes the need to subjectively select the change point in the time series and calculate the intervention impact accordingly. The utility of our approach is demonstrated via simulation studies and real data with strong and known vaccine effects.

## ACKNOWLEDGMENTS

The authors would like to thank Gerardo Chowell for his help in identifying appropriate data sets for analysis.

## Supplementary Material
