# Supplementary material for: Bayesian Model Averaging with Change Points to Assess the Impact of Vaccination and Public Health Interventions
Source: Epidemiology. 2017 Sep 28;28(6):889–97. doi: 10.1097/EDE.0000000000000719 (PMC5617796; doi:10.1097/EDE.0000000000000719)
Supplement: Supplementary file 1 [file ede-28-889-s001.pdf]

Bayesian model averaging with change points to assess the impact of vaccination and public health interventions

SUPPLEMENTARY METHODS

Data sources

U.S. hospitalization data were obtained from the Healthcare Cost and Utilization Project (HCUP) State Inpatient Databases (SID) for the period 1996 through 2010 for 10 states: Arizona, Colorado, Iowa, Massachusetts, New Jersey, New York, Oregon, Utah, Washington, and Wisconsin. The SID contain ~100% samples of ICD9-coded (international classification of diseases, ninth revision) hospitalization data for these states [1].

Data on hospitalizations in Brazil were obtained for the period 2003 through 2013 from the Brazil Unified Health System (Sistema Único de Saúde; SUS), which maintains a nationwide administrative database that records all hospitalizations paid by the public sector; the data we used are available to the public through SUS and were obtained from the Ministry of Health. The database includes ICD10-coded hospitalizations from government-owned hospitals, as well as private and non-profit hospitals under contract to the SUS.

Data on hospitalizations in Chile were obtained for the period 2001 through 2012 from the Chilean Ministry of Health, Department of Statistics and Health (Departamento de Estadísticas e información de Salud; DEIS) information. Hospitals in Chile are required to submit ICD-10 coded (international classification of diseases, tenth revision) diagnostic, patient demographic and other data on all hospitalizations to the DEIS, which aggregates these data and publishes it online [2].

In the U.S., PCV coverage of all infants <1 year was above 80% after 12 months, and in Brazil and Chile, with centrally directed health delivery systems, it was well above 90%.

Model averaging, specification of priors, and calculation of posteriors

For the Bayesian model averaging, we fit each of the three model structures with every possible combination of covariates and each candidate change point. This results in a large set of candidate models. For each of these, the Bayesian information criterion (BIC) [3] was estimated, which measures the goodness of the model fit while penalizing more complex structures. These BIC scores were used, along with the prior probabilities for each model, to assign a weight (posterior probability) to each model. To estimate the model weights (posterior probabilities), we used the approach of Burnham and Anderson (2004)[4]. Based on the BIC scores, we calculate the posterior probability/weight for each model by

$$p_i = \frac{\exp\left(-\frac{1}{2}\Delta BIC_i\right)q_i}{\sum_{r=1}^{R}\exp\left(-\frac{1}{2}\Delta BIC_r\right)q_r},$$

where ΔBIC_i is the difference of BIC for model *i* and the minimum BIC value among all models, and q_i is the prior probability of model *i*, where i=1 to R, the total number of models.

For Bayesian methods, selecting a suitable prior probability is important. We followed a weakly informative approach when assigning priors, assuming first that it was equally likely that a change point did or did not exist, and second that if a change point did exist, that it was equally likely to be located at any particular time point. Likewise,

we used non-informative priors for additional covariates, with an equal prior probability that the model includes a particular variable.

After computing the weight of each model, we computed the model-averaged regression coefficients and their corresponding variances as

$$\hat{\hat{\beta}} = \sum_{i=1}^{R} \hat{\beta}_i w_i,$$

$$\widehat{\mathrm{var}}\left(\hat{\hat{\beta}}\right) = \sum_{i=1}^{R} w_i \left[\widehat{\mathrm{var}}\left(\hat{\beta}_i\right) + (\hat{\beta}_i - \hat{\hat{\beta}})^2\right],$$

where $\hat{\beta}_i$ is the estimate for the parameter and $\widehat{\mathrm{var}}\left(\hat{\beta}_i\right)$ is its variance in model i.

Finally, we estimated the magnitude of change in an outcome by comparing model-averaged fitted values with counterfactual predicted values. We computed model-averaged fitted values as

$$\hat{Y} = \sum_{i=1}^{R} w_i \hat{Y}_i \,,$$

where $\hat{Y}_i$ is the fitted value under model i and w_i is the posterior probability of this model. The weighted average of the predicted value from each of the candidate models provides a consensus estimate of the predicted value at each time point. The corresponding estimated variance for the model-averaged fitted values is $\widehat{\mathrm{var}}(\hat{Y}) = \sum_{i=1}^{R} w_i \{\widehat{\mathrm{var}}(\hat{Y}_i)\}$, where $\{\widehat{\mathrm{var}}(\hat{Y}_i)\}$ is the estimated variance for the fitted value under model i. Thus, the 95% approximate pointwise confidence interval for the model-averaged fitted values is computed as

$$\hat{Y} \pm 1.96 \left\{\widehat{\mathrm{var}}(\hat{Y})\right\}^{1/2}.$$

Estimation of the smooth function $g(t_j)$*:*

For the sake of simplicity, we described the estimation procedure in terms of linear mixed models; extension to generalized mixed models with change points is straightforward. The procedure given below describes the estimation of the smooth function $g(t_j)$ (equations (1)-(3) in the main text) via a nonparametric mixed model approach. By using this approach, the smooth function is decomposed into fixed and random effects.

The observations $y_j, j = 1, \ldots, K$ at times t_j follow the relationship

$$y_j = g(t_j) + \varepsilon_j, \qquad \text{(S1)}$$

where $t_1 < \cdots < t_K$, $g(\cdot)$ is an unknown smooth function to be estimated, and the error term follows a normal distribution with mean zero and covariance matrix $\sigma^2\mathbf{R}$**.** The smoothing spline for fitting a function of the form (S1) in the one dimensional case is computed by maximizing the following penalized likelihood[5]

$$\text{penalized likelihood} = \log \text{likelihood} - \lambda \int g''(t)^2 dx\,, \qquad \text{(S2)}$$

where λ_k controls the amount of smoothing.

Let $\boldsymbol{\mathcal{Q}}$ *and* $\boldsymbol{\mathcal{G}}$ to be $K \times (K-2)$ and $(K-2) \times (K-2)$ matrices, respectively, where

$$\boldsymbol{\mathcal{Q}}_{ii} = \frac{1}{h_i}, \mathcal{Q}_{i+1,i} = -\left(\frac{1}{h_i} + \frac{1}{h_{i+1}}\right), \boldsymbol{\mathcal{Q}}_{i+2,i} = \frac{1}{h_{i+1}},$$

$$\boldsymbol{\mathcal{G}}_{i,i+1} = \frac{h_{i+1}}{6}, \boldsymbol{\mathcal{G}}_{ii} = \frac{h_i + h_{i+1}}{3},$$

are the only nonzero elements in these matrices with $h_j = t_{j+1} - t_j$, and $j, i = 1, \ldots, K-1$.

At the observed data points, the cubic smoothing spline estimate for g is

$$\tilde{\mathbf{g}} = (\mathbf{R}^{-1} + \lambda \boldsymbol{\mathcal{Q}}\boldsymbol{\mathcal{G}}^{-1}\boldsymbol{\mathcal{Q}}^{T})^{-1}\mathbf{R}^{-1}\mathbf{y}, \qquad \text{(S3)}$$

where $\mathbf{y} = (y_1, \ldots, y_K)^T$.

Define $\mathbf{U}$ to be a $\mathrm{K} \times 2$ matrix whose first column is a vector 1s and the second column is

$(\mathrm{t}_1, \ldots, \mathrm{t}_{\mathrm{K}})^{\mathrm{T}}$ and $\mathbf{Z} = \boldsymbol{\mathcal{Q}}(\boldsymbol{\mathcal{Q}}^{\mathrm{T}}\boldsymbol{\mathcal{Q}})^{-1}$. According to [5], equation (S3) can be written as follows:

$$\tilde{\mathbf{g}} = \mathbf{U}^{\mathrm{T}}\,\hat{\boldsymbol{\varphi}} + \mathbf{Z}^{\mathrm{T}}\hat{\boldsymbol{\gamma}}, \tag{S4}$$

where $\tilde{\mathbf{g}}$ is the best linear unbiased predictor of a conditional mean vector (See [5] for the technical proof). Given equation (S4), (S1) can be written as a mixed model

$$\mathbf{y} = \mathbf{U}^{\mathrm{T}}\,\boldsymbol{\varphi} + \mathbf{Z}^{\mathrm{T}}\boldsymbol{\gamma} + \boldsymbol{\varepsilon}, \tag{S5}$$

where $\boldsymbol{\varphi}$ is the 2×1 vector of fixed effects, $\boldsymbol{\gamma}$ are the normally-distributed $(\mathrm{K} - 2) \times 1$ vector of random effects with mean zero and covariance matrix $\boldsymbol{\mathcal{D}} = \boldsymbol{\mathcal{G}}\sigma^2$, and ε are normally distributed error terms with mean zero and variance σ^2. In the penalized likelihood (S2), the log likelihood is the conditional likelihood of $\mathbf{y}$ given $\boldsymbol{\gamma}$, and the penalty function is proportional to the log-density function of $\boldsymbol{\gamma}$.

In order to estimate the random effects, first, we transformed the random effects to independent random effects by $\boldsymbol{\gamma} = \mathbf{L}\boldsymbol{\delta}$, and $\mathbf{Z}'^{\mathbf{T}} = \mathbf{Z}^{\mathrm{T}}\mathbf{L}$, where L is the lower triangle of the Cholesky decomposition of $\boldsymbol{\mathcal{G}}$. Then (S5) can be reformulated as

$$\mathbf{y} = \mathbf{U}^{\mathrm{T}}\boldsymbol{\varphi} + \mathbf{Z}'^{\mathbf{T}}\boldsymbol{\delta} + \boldsymbol{\varepsilon},$$

where $\boldsymbol{\delta} \sim \mathrm{N}(0, \mathbf{I}\sigma^2)$.

The R package gamm4 can be used to implement this type of random effects while fitting generalized linear mixed models. Running a single dataset containing 120 months with 120 candidate change point locations and a single covariate took 87 seconds when implemented with a 3.5 GHz Intel Core i7 CPU.

As we used observational-level random effects, using traditional methods to estimate random effects run out of degrees of freedom, thus, using the smoothing splines approach

was useful to avoid this challenge. In addition, this approach takes the temporal correlations into account and adjusts for the unobserved trends in the data by using the splines. However, a comparison of this method to mixed models with random effects that has a different covariance structure such as lag 1 autocorrelation (AR(1)) would be an interesting area for future work.

Characteristics of bootstrap samples

As the original bootstrap proposed by [6] is for iid random samples, it cannot be directly applied to dependent data. Therefore, to estimate the distribution of the incidence rate ratio, we proposed to use a nonparametric bootstrap method, which suggest applying the classical bootstrap method to the residuals.

Let $Y_1, \dots, Y_n$ be the time series observations. For some fixed $p \in \mathbb{N}$, denote the estimator of the conditional expectation $E(Y_j|Y_{j-1}, \dots, Y_{j-p})$ by $\widehat{m}_n(Y_{j-1}, \dots, Y_{j-p})$. This estimator results in the following residuals:

$$\hat{e}_j \coloneqq Y_t - \widehat{m}_n\left(Y_{j-1}, \dots, Y_{j-p}\right), j = p + 1, \dots, n,$$

and in the next step, we calculate the bootstrap time series as follows

$$Y_j^* = \widehat{m}_n\left(Y_{j-1}, \dots, Y_{j-p}\right) + e_j^*, j = 1., \dots, n,$$

where $e_1^*, \dots, e_n^*$ the residuals sampled from $\{e_1, \dots, e_n\}$ with replacement. An alternative to the above equation would be to use $\widehat{m}_n\left(Y_{j-1}^*, \dots, Y_{j-p}^*\right)$ instead of $\widehat{m}_n\left(Y_{j-1}, \dots, Y_{j-p}\right)$, but as pointed out by [7] using $\widehat{m}_n\left(Y_{j-1}, \dots, Y_{j-p}\right)$ creates the stability and satisfies some weak dependence properties for the triangular array of dependent observations that helps establishing asymptotic consistency along with other asymptotic results of the bootstrap process. We obtain the estimator $\widehat{m}_n\left(Y_{j-1}, \dots, Y_{j-p}\right)$ using the models built for Brazil,

Chile, and the U.S. data sets, respectively. After generating 400 bootstrap samples, we run our estimation procedure and obtain the incidence rate ratio for each sample.

Note that estimation of the bootstrap confidence intervals for the IRR can be computationally heavy, a naïve approach to obtain the confidence intervals is by dividing the upper and lower bounds of the confidence interval of the model-averaged fitted values by the counterfactual predictions. The statistical significance of IRR obtained from the naïve approach is same as the bootstrap approach, and the upper and lower bounds would be close.

Characteristics of simulated data sets

We generated five sets of simulated time series that resembled observed time series in terms of number of monthly cases, seasonality, and degree of random unexplained variability but on which we imposed changes of known timing and magnitude. Specifically, for each set we generated 100 time series that followed a Poisson distribution given by

$$Y_i \sim \text{Poisson}(\mu_i)$$

$$\log(\mu_i) = \log(N) + \kappa * t_i + \delta * h(t_i) + (t_i - \theta) * \eta, \qquad (4)$$

where N is the number of all cause hospitalizations per month, κ is the increase in number of cases per year unrelated to the vaccine, δ is the seasonal amplitude , $h(t_i)$ is the harmonic term (calculated by $\cos(2 * \pi * t_i/12 + \pi)$), θ is the change point month, η is the vaccine-associated change per year (given by $\log(1 + (\nu/100))/12$ where ν as the vaccine-associated decline/year), and $i = 1,2, \dots, n$ with n as the total number of time points. The parameters used to generate the simulations in equation (4) were extracted

from IPD and pneumonia time series from the U.S., Chile, and Brazil using a Poisson regression model in PROC MCMC in SAS[8]. The last simulation study used parameters obtained from Brazil pneumonia series and was used to demonstrate the performance of our method in the absence of a vaccine effect.

In the U.S. IPD simulation, each time series had 4 years of pre-vaccine and 10 years of post-vaccine data, with an average of 16 cases per month, and change points at 06/2000 and 12/2003. We evaluated vaccine-associated rate reduction of 10% per year for 3.5 years beginning in 6/2000 and allowed the vaccine effect to be constant after 12/2003. In the U.S. ACP simulation, each time series had the same amount of data as in the first simulation, but this time an average of 650 cases per month. In this simulation, we imposed two change points: 12/1997 and 01/2004. Starting from the first change point, we assessed a vaccine effect of 3% decline per year until the second change point and allowed the vaccine effect to be constant after the second change point. In the third simulation, the time series mimicked the Chilean all-cause pneumonia data by having 10 years of pre-vaccine and 1 year of vaccine data with an average of 118 cases per month. In this simulation, we introduced two change points: 07/2007 and 07/2011, and imposed a vaccine effect of 3% decline per year between these points. In the fourth and fifth simulation studies, each data set had 7 and 3 years of pre-vaccine and post-vaccine data, respectively, with 10000 cases per month replicating the Brazil all-cause pneumonia data. In the fourth study, we introduced a change point in 07/2010, and used a vaccine effect of 3% per year until 12/2013. We imposed a vaccine effect of 0% in the last simulation study to demonstrate the performance of our approach in the absence of a vaccine effect.

Comparison of traditional interrupted time-series approach with BMA-CP

For the simulated data sets, in the ITS approach, we used an autoregressive model of order 1, which includes terms for the vaccine introduction and secular trend, along with harmonic terms with 6-and 12-month periods to account for seasonality. The comparison of the BMA-CP approach with ITS (Supplementary Table 2) shows that for a data set with a single change point (similar to characteristics of Brazil ACP data), these two methods give mostly comparable results. One exception is when the ITS had a cut off point 12 months after the true change point, we observed that ITS estimated an IRR of 1, whereas the true IRR is 0.970 for the simulated data set with 3% vaccine effect. For the simulation with two change points (similar to characteristics of Chile ACP data), the IRR results from the ITS approach were generally more biased than the BMA-CP approach. With two change points, the results of ITS and BMA-CP were closest when the cut off for ITS was close to the mean second change point calculated with BMA-CP (Table 1).

In the data applications, in our ITS analysis, we used an autoregressive model, in which, error covariance structures had lag 1 autocorrelation (AR(1)), and we included terms for the vaccine introduction, secular trend and an interaction between these terms along with harmonic terms with 6-and 12-month periods to account for seasonality. In Brazil ACP data, the results from the two models were comparable when the ITS cut off was close to the change point indicated by the BMA-CP approach (**Figure 4**). However, with the Chile data, ITS approach gives results larger than the BMA-CP approach except

for 12 to 23 months olds when the cut off is at the vaccine introduction. Note that with the Chile data, the amount of data after the vaccine introduction was limited.

Tables

Supplementary Table 1: Definitions

Outcome		ICD9 codes (U.S.)	ICD 10 codes (Brazil, Chile)
Invasive pneumococcal disease (IPD)		Any mention of 320.1 OR 038.2 OR [(320.8 OR 790.7 OR 038.9 OR 995.91 OR 995.92) AND 041.2]	---
Pneumococcal (lobar) pneumonia		481	---
All-cause pneumonia (ACP), Standard definition		Any mention of 480-486	J12-18
All-cause pneumonia (ACP), definition of Griffin et al*		First listed pneumonia (480-486 OR 487.0) OR [first listed meningitis, septicemia or empyema AND any mention of pneumonia (480-486 OR 487.0)]	--
	Meningitis	321.xx, 013.x, 003.21, 036.0, 036.1, 047, 047.0, 047.1, 047.8, 047.9, 049.1, 053.0, 054.72, 072.1, 091.81, 094.2, 098.82, 100.81, 112.83, 114.2, 115.01, 115.11, 115.91,130.0, 320, 320.0, 320.1, 320.2, 320.3, 320.7, 320.81, 320.82, 320.89, 320.8, 320.9, 322, 322.0, 322.9	---
	Septicemia	038.1x, 038.4x, 003.1, 020.2, 022.3, 031.2, 036.2, 038, 038.0, 038.2, 038.3, 038.8, 038.9, 054.5, 785.52, 790.7, 995.91, 995.92	---
	Empyema	510	---
Influenza		487	J09-J11
Rotaviral enteritis		008.61	---

Urinary tract infection (UTI)	599.0	N39.0

Supplementary Table 2. Results of simulations.

Simulated data has characteristics similar to:	Average cases/month	Number of months before the first change point, between the change points and after the second change point**	True IRR 12 months after the first/second change point*	Median IRR +/- range of 2.5, 97.5 percentiles of 100 simulations 12 months after the first/second change point*	Percent of simulation that detect a change (IRR<1)	True change point	Mean change point
U.S. data, IPD	16	53, 42, 73	0.691	0.703 (0.639, 0.778)	100	54, 96	59.8, 97.1
U.S. data, ACP	650	23, 73, 72	0.830	0.837 (0.788, 0.893)	100	24, 97	35.7, 98.5
Brazil data, ACP	10000	84, 48	0.970	0.948 (0.890, 0.976)	98	91	91.8, ***
Chile data, ACP	1180	78, 48, 18	0.889	0.898 (0.739, 0.992)	97	79, 127	83, 112.5

Supplementary Table 3. Comparison of IRR values calculated using interrupted time series (ITS) and Bayesian model averaging with change points (BMA-CP) approaches for simulated data

Simulated data has characteristics similar to:	Average cases/month	Change point location(s)	True IRR 12 months after the first/second change point*	Cut off month for ITS	Median IRR +/-range of 2.5, 97.5 percentiles of 100 simulations 12 months after the change point*	
					ITS	BMA-CP
Brazil data, ACP	10000	85	0.970	79	0.941 (0.899, 0.986)	0.948 (0.890, 0.976)
				85	0.953 (0.926, 0.990)	
				91	0.976 (0.957, 0.993)	
				97	1.000 (1.000, 1.000)	
Brazil data, ACP with 0% vaccine effect	10000	85	1.000	79	0.999 (0.941, 1.062)	0.995 (0.973, 1.001)
				85	0.999 (0.960, 1.044)	
				91	0.999 (0.978, 1.023)	
				97	1.000 (1.000, 1.000)	
Chile data, ACP	1180	79, 127	0.889	73	0.792 (0.569, 1.207)	0.898 (0.739, 0.992)
				79	0.795 (0.585, 1.199)	
				91	0.823 (0.619, 1.190)	
				115	0.873 (0.673, 1.172)	
				127	0.921 (0.718, 1.184)	
				139	1.000 (1.000, 1.000)	

Supplementary Table 4. Comparison of estimated percent declines (1-IRR)*100) calculated using interrupted time series (ITS) and Bayesian model averaging with change points (BMA-CP) approaches for Brazil and Chile ACP hospitalizations data

Data set	Age group	Cut off month*	Percent decline 24 months after vaccine	Percent decline 48 months after vaccine

			introduction [95%CI]		introduction [95%CI]	
Brazil			ITS	BMA-CP	ITS	BMA-CP
		85	14% (11%, 17%)		18% (15%, 21%)	
	0 to <12	97	10% (6%, 13%)	9% (3%, 14%)	9% (6%, 12%)	10% (4%, 19%)
		109	0% (-4%, 4%)		3% (-1%, 6%)	
		85	14% (10%, 17%)		20% (17%, 23%)	
	12 to 23	97	13% (9%, 17%)	6% (1%, 9%)	13% (9%, 16%)	7% (1%, 10%)
		109	0% (-5%, 5%)		3% (-1%, 7%)	
		85	11% (7%, 15%)		20% (16% 23%)	
	24 to 59	97	10% (7%, 14%)	9% (3%, 11%)	14% (11%, 18%)	11% (4%, 13%)
		109	0% (-4%, 4%)		5% (1%, 9%)	
Chile	0 to <12	121	19% (7%, 30%)	9% (1%, 17%)		
		133	59% (53%, 65%)			
	12 to 23	121	9% (-11%, 25%)	18% (4%, 26%)		
		133	43% (32%, 53%)		—	—
	24 to 59	121	21% (8%, 32%)	5% (-1%, 6%)		
		133	34% (24%, 43%)			

Supplementary Table 5. Estimated percent decline (1-IRR)*100) and probabilities that changes occurred after vaccination by age group, country, and outcome

Outcome/age group	Percent decline 24 months after vaccine introduction [95%CI]	Percent decline 48 months after vaccine introduction [95%CI]	Probability of any change in the time	Probability that the change occurred

				series	after vaccine introduction*
Rotaviral enteritis					
	0 to <12 (U.S.)	1% (-10%, 5%)	2% (-10%, 4%)	1.000	1.000
Urinary tract infection					
	0 to <12 (U.S.)	10% (4%, 13%)	23% (13%, 24%)	1.000	0.999
	0 to <12 (Brazil)	-4% (-10%, 2%)	-5% (-8%, 1%)	1.000	0.004
	12 to 23 (U.S.)	7% (2%, 9%)	7% (1%, 10%)	0.880	0.718

Figures

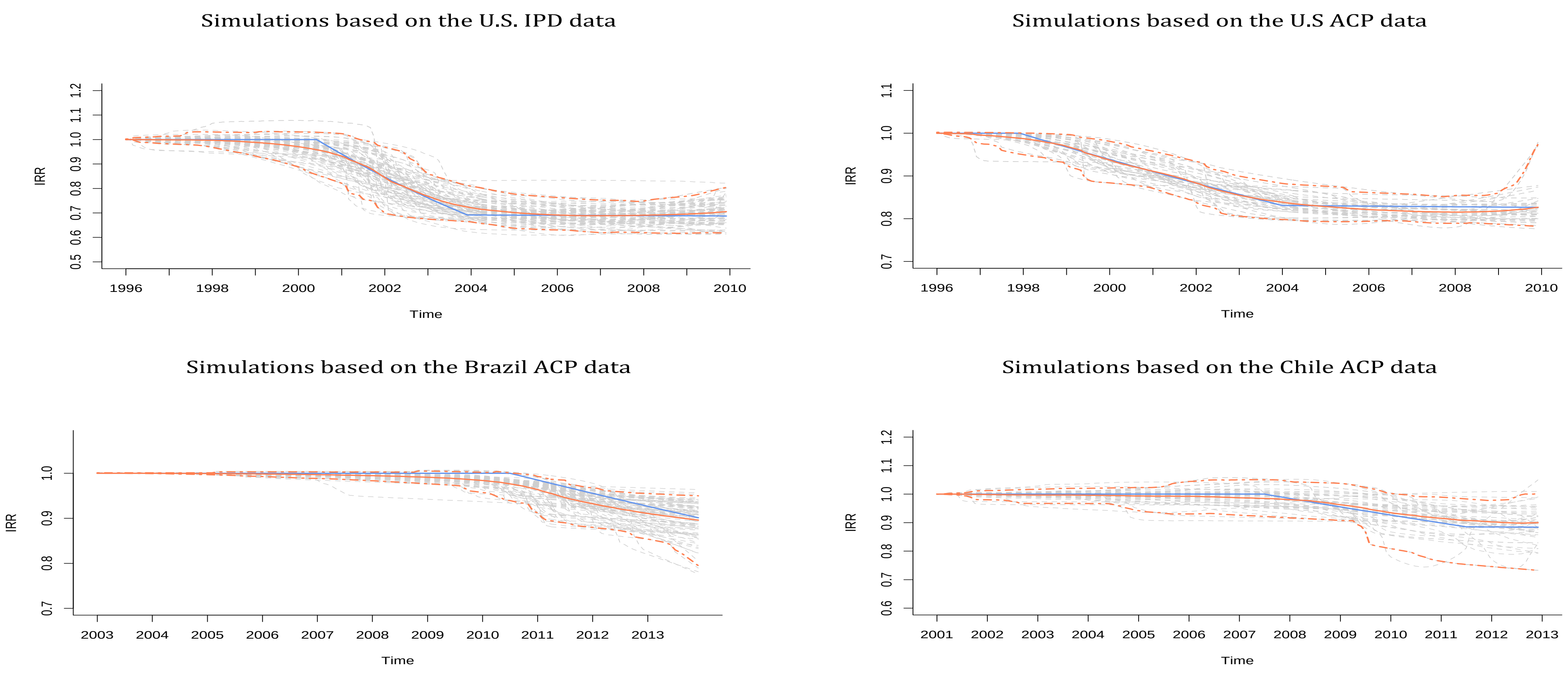

Supplementary Figure 1. The estimated incidence rate ratios (gray dashed line) from each of 100 simulation runs along with 2.5 and 97.5 percentiles (orange dashed-dotted lines) based on 100 simulation runs. The simulated data are based on the time series characteristics of the indicated country and disease. The blue and orange solid lines represent the true IRR value and the median estimated IRR, respectively, at each time point.

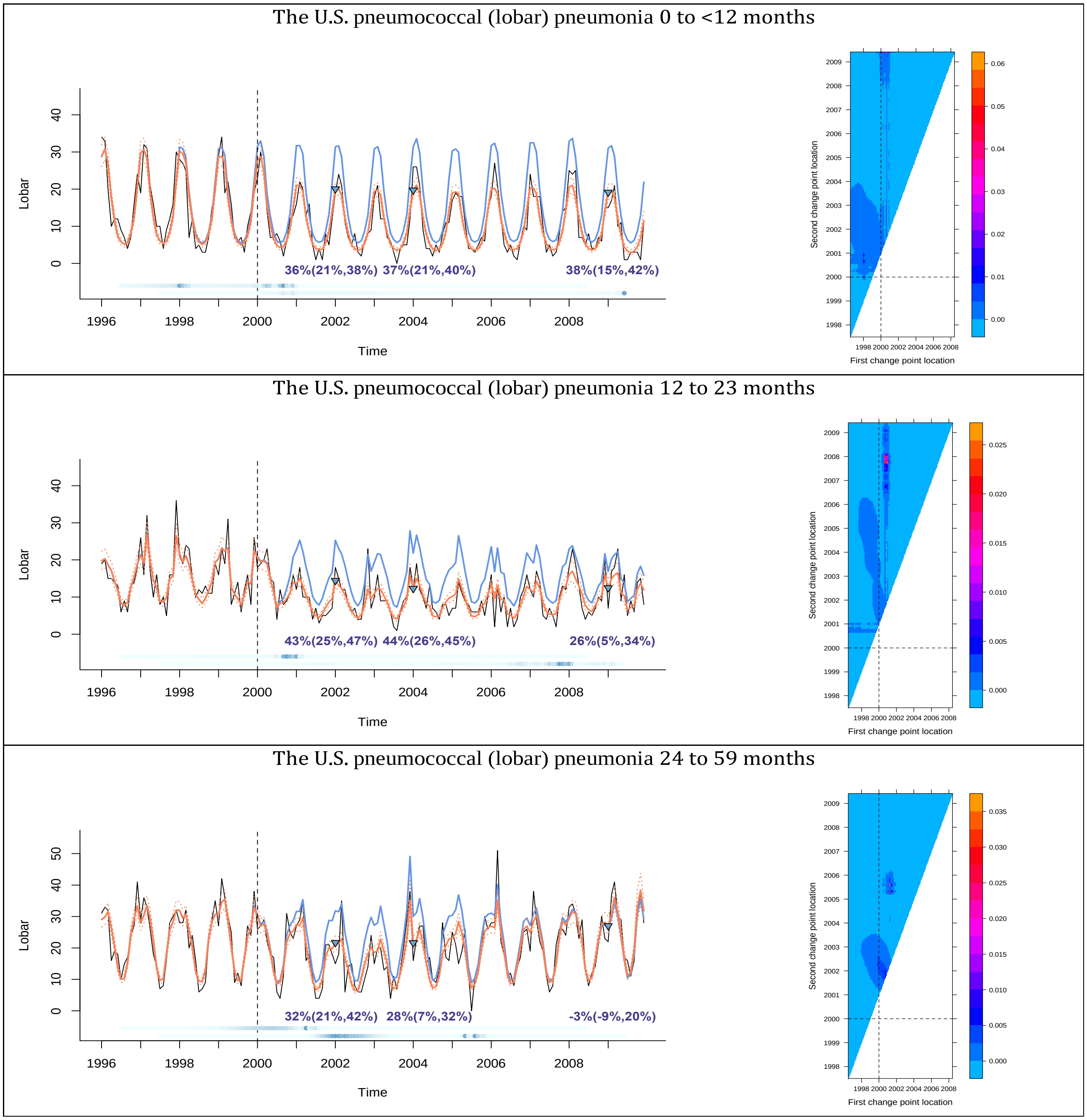

Supplementary Figure 2. (A) Pneumococcal (lobar) pneumonia hospitalizations versus time for 10 U.S. states by age group, showing observed pneumococcal (lobar) pneumonia hospitalizations per month (black), model-averaged fitted values (orange, solid) with their 95% approximate pointwise confidence intervals (orange, dotted) and counterfactual predicted values (blue). The estimated decline at specific time points (green triangles) are shown, with their respective 95% bootstrap confidence intervals. (B) Posterior probabilities corresponding to the plots in (A) for the locations of the first (x-axis) and second (y-axis) change points. The dashed lines represent the time that the PCV7 (01/2000) is introduced. The blue dots at the bottom represent the probability of a change occurring at that point. The color gets darker as the probability increases. The first and second sets of dots are for the first and second change points, respectively.

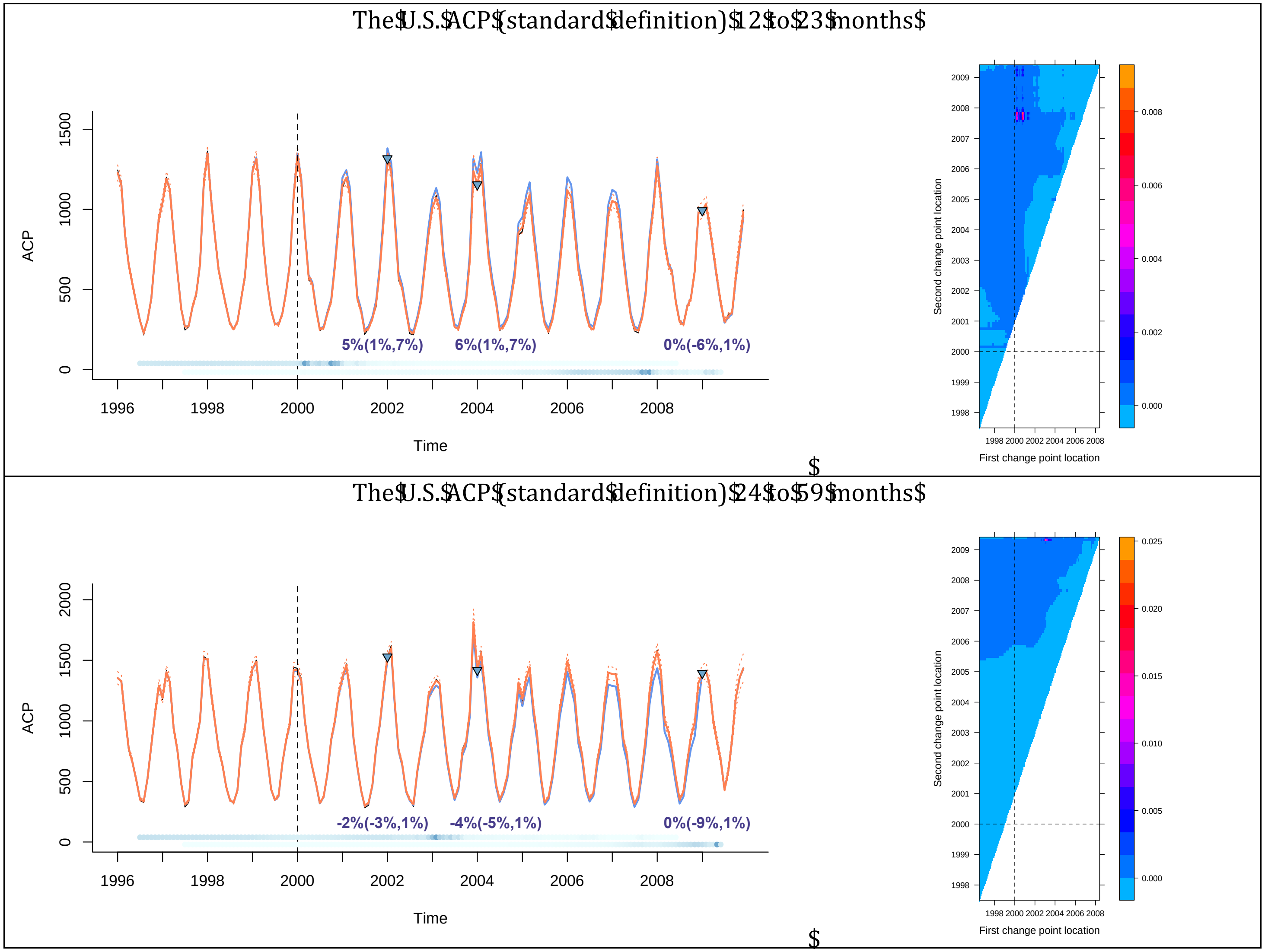

Supplementary Figure 3. (A) ACP (standard definition) hospitalizations versus time for 10 U.S. states by age group, showing observed ACP hospitalizations per month (black), model-averaged fitted values (orange, solid) with their 95% approximate pointwise confidence intervals (orange, dotted) and counterfactual predicted values (blue). The estimated decline at specific time points (green triangles) are shown, with their respective 95% bootstrap confidence intervals. The blue dots at the bottom represent the probability of a change occurring at that point. The color gets darker as the probability increases. The first and second sets of dots are for the first and second change points, respectively. (B) Posterior probabilities corresponding to the plots in (A) for the locations of the first (x-axis) and second (y-axis) change points. The dashed lines represent the time that the PCV7 is introduced.

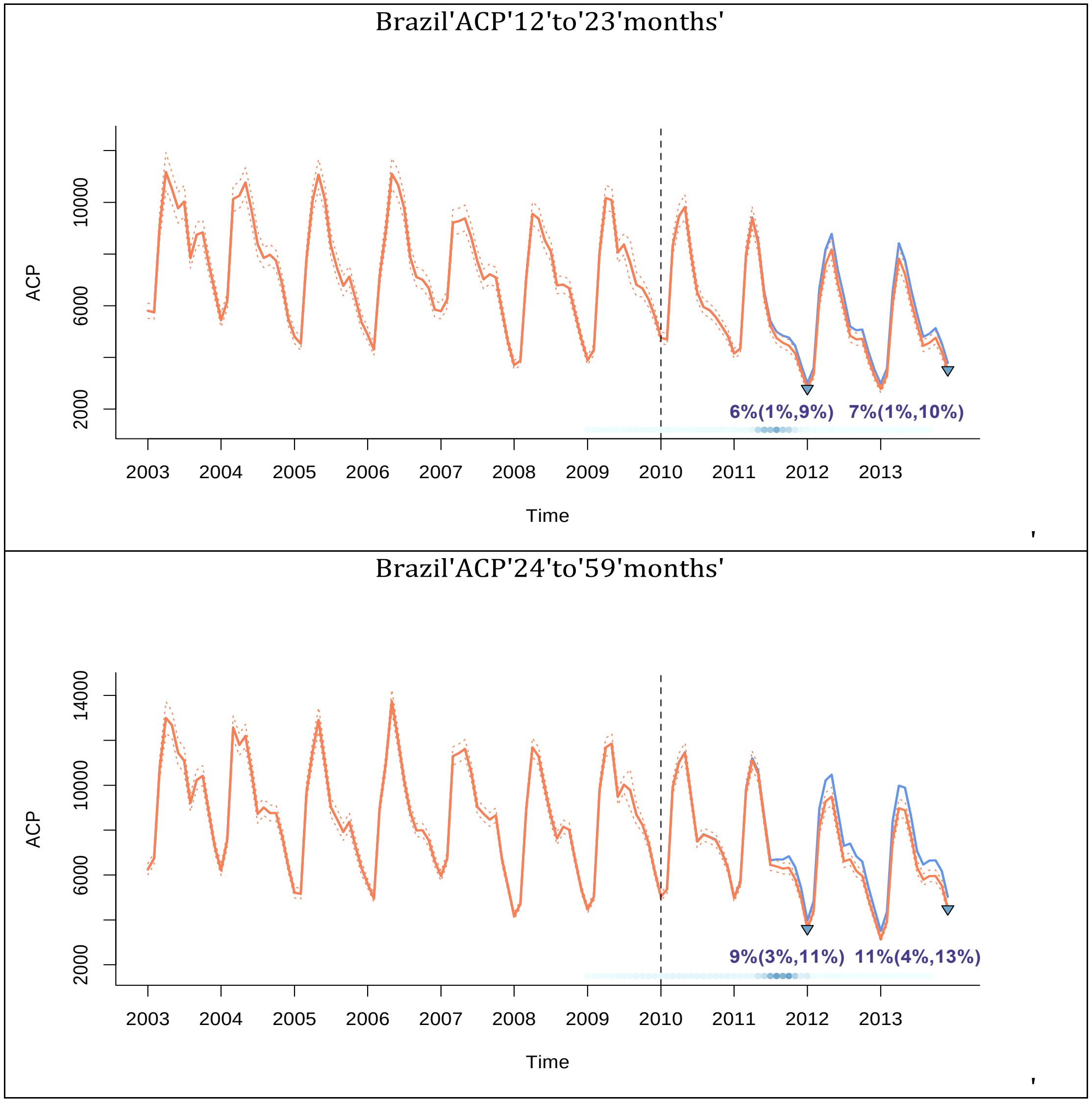

Supplementary Figure 4. ACP hospitalizations versus time in Brazil by age group, showing observed ACP hospitalizations per month (black), model-averaged fitted values (orange, solid) with their 95% approximate pointwise confidence intervals (orange, dotted) and counterfactual predicted values (blue). The estimated decline at specific time points (green triangles) are shown, with their respective 95% bootstrap confidence intervals. The blue dots at the bottom represent the probability of a change occurring at that point. The color gets darker as the probability increases.

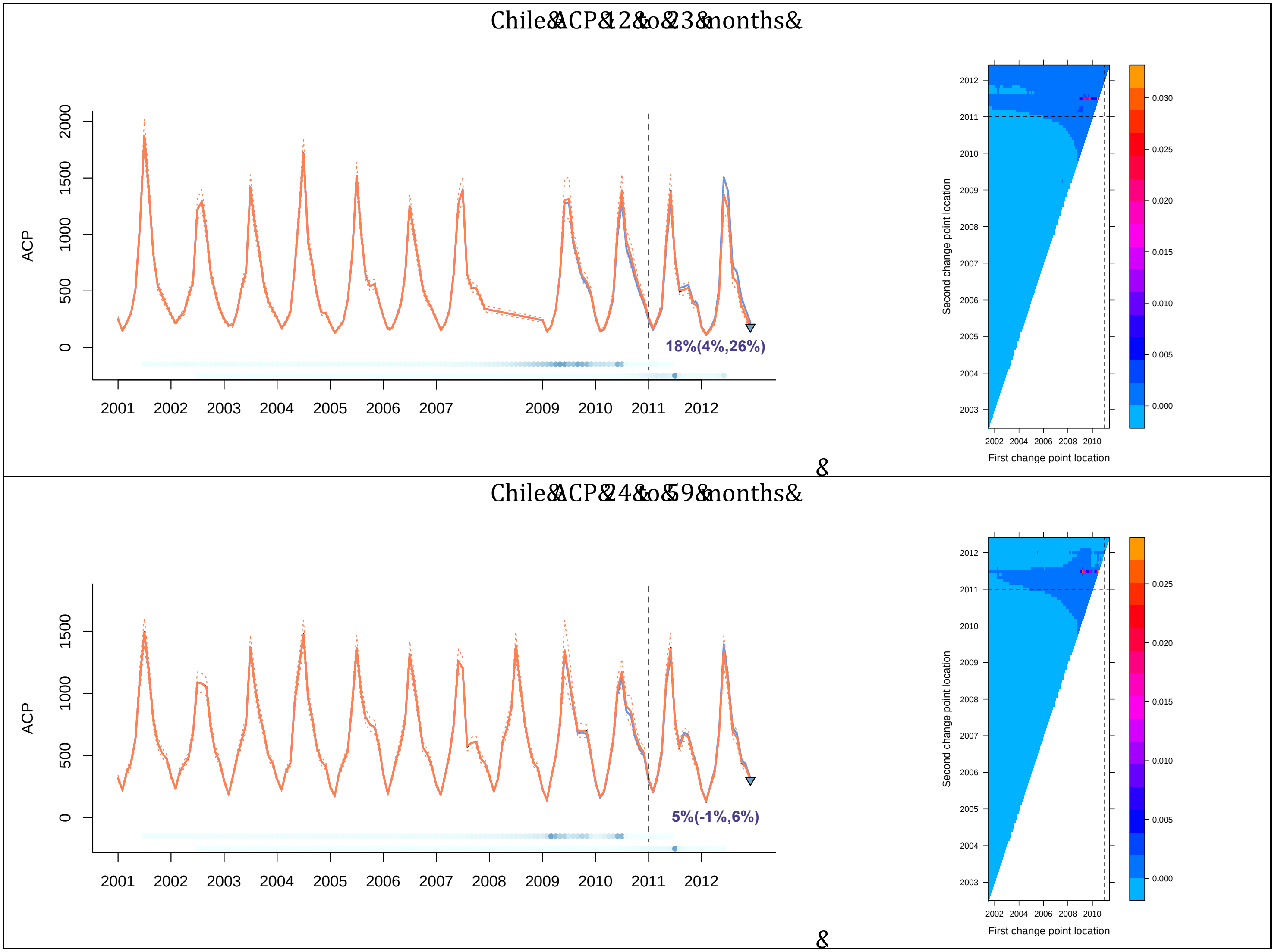

Supplementary Figure 5. (A) ACP hospitalizations versus time in Chile by age group, showing observed ACP hospitalizations per month (black), model-averaged fitted values (orange, solid) with their 95% approximate pointwise confidence intervals (orange, dotted) and counterfactual predicted values (blue). The estimated decline at specific time points (green triangles) are shown, with their respective 95% bootstrap confidence intervals. The blue dots at the bottom represent the probability of a change occurring at that point. The color gets darker as the probability increases. The first and second sets of dots are for the first and second change points, respectively. (B) Posterior probabilities corresponding to the plots in (A) for the locations of the first (x-axis) and second (y-axis) change points. The dashed lines represent the time that the PCV10 is introduced.

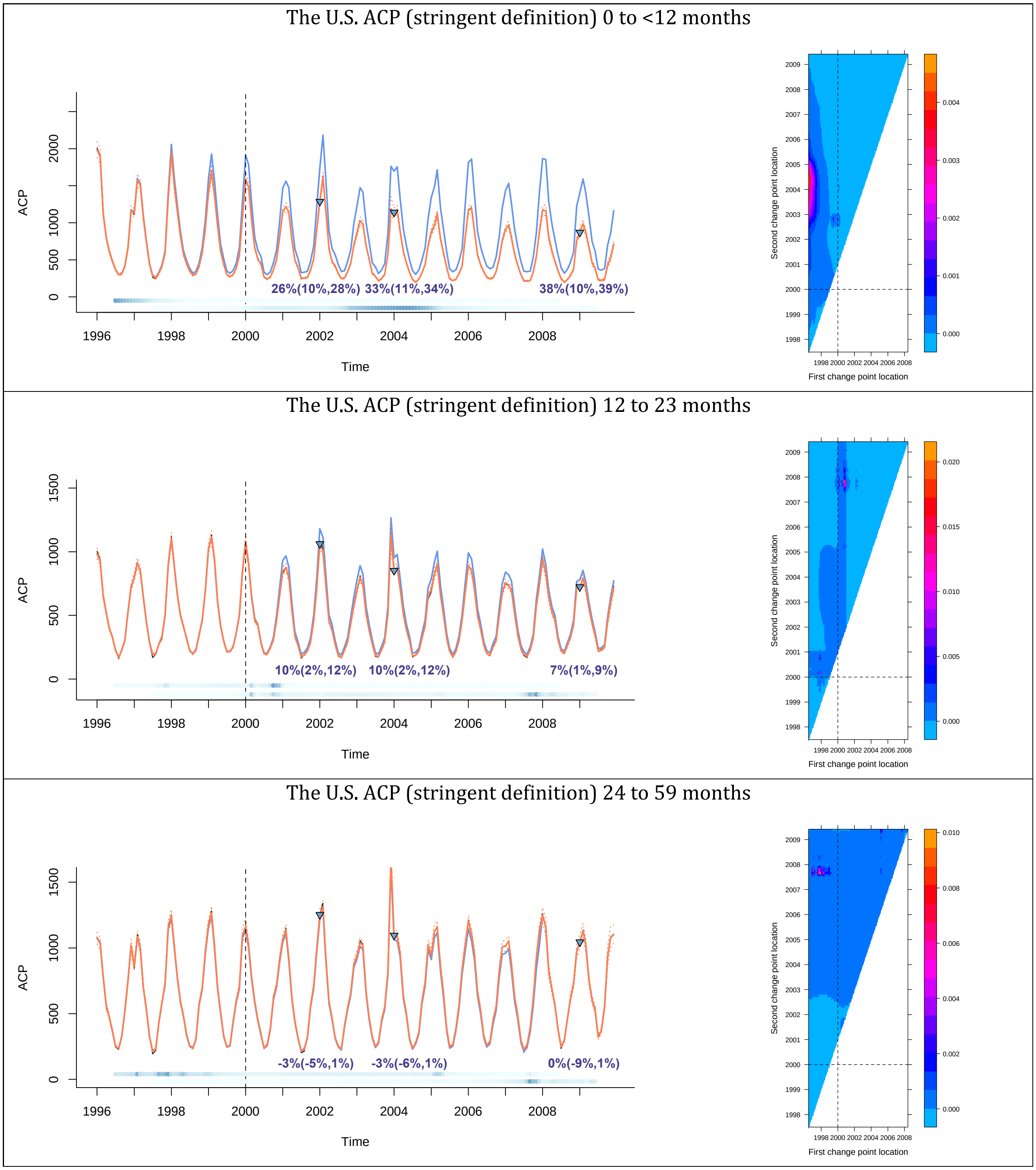

Supplementary Figure 6. (A) ACP (stringent definition) hospitalizations versus time for 10 U.S. states by age group, showing observed ACP hospitalizations per month (black), model-averaged fitted values (orange, solid) with their 95% approximate pointwise confidence intervals (orange, dotted) and counterfactual predicted values (blue). The estimated decline at specific time points (green triangles) are shown, with their respective 95% bootstrap confidence intervals. (B) Posterior probabilities corresponding to the plots in (A) for the locations of the first (x-axis) and second (y-axis) change points. The dashed lines represent the time that the PCV7 (01/2000) is introduced. The blue dots at the bottom represent the probability of a change occurring at that point. The color gets darker as the probability increases. The first and second sets of dots are for the first and second change points, respectively.

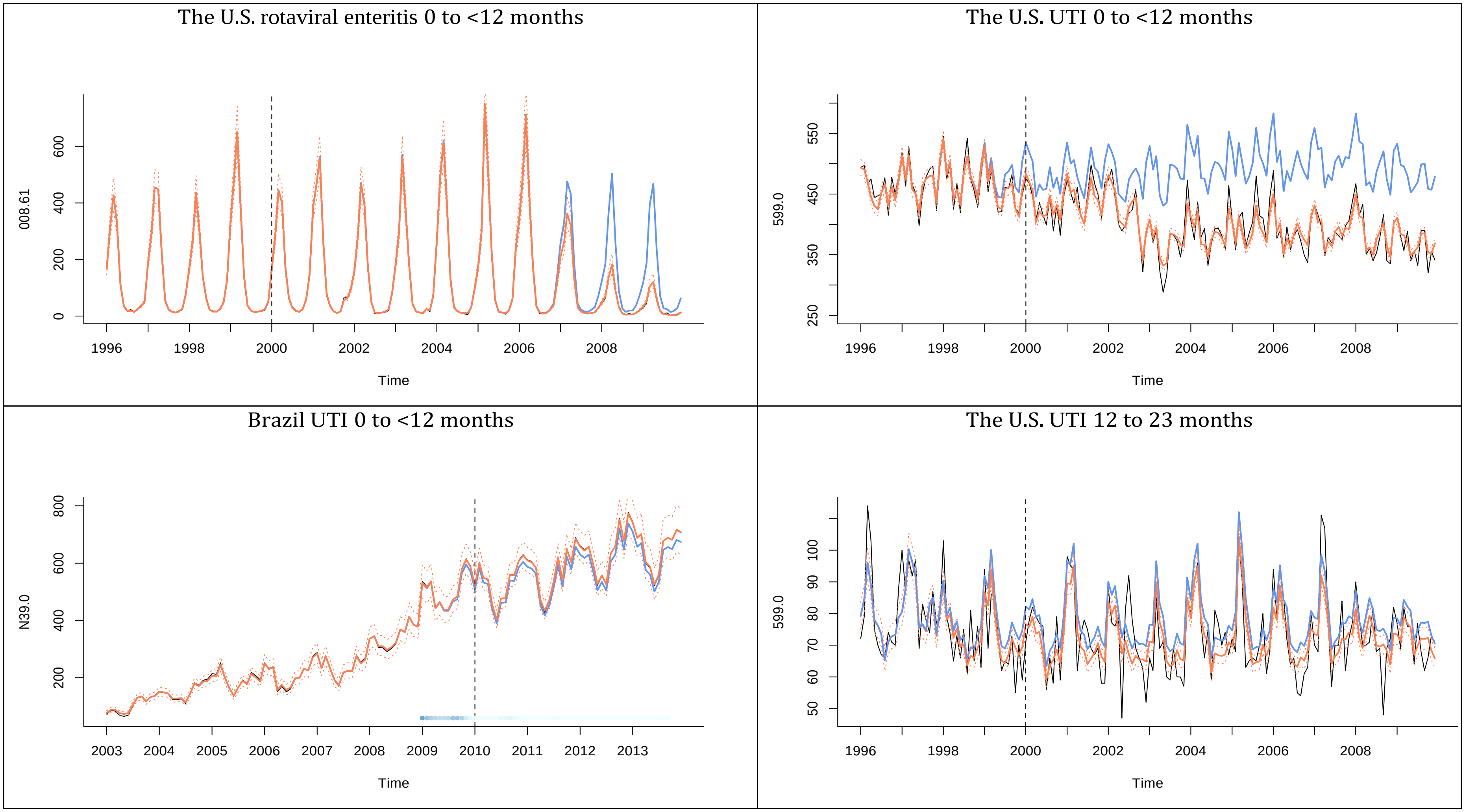

Supplementary Figure 7. Non-pneumoccocal outcomes. Rotaviral enteritis hospitalizations and UTI versus time. The black line indicates hospitalizations per month. The blue solid line shows the counterfactual predicted values. The orange solid and dashed lines show the model-averaged fitted values and their 95% approximate pointwise confidence intervals, respectively.

References

1. Healthcare Cost and Utilization Project (HCUP). Overview of the State Inpatient Databases (SID). Rockville, MD: Agency for Healthcare Research and Quality; 2014, accessed on June, 2011. Available at: http://www.hcup-us.ahrq.gov/sidoverview.jsp.
2. Chilean Ministry of Health DoSaH, accessed on September, 2014. http://deis.minsal.cl/BDPublica/BD_Egresos.aspx.
3. Schwarz G. Estimating the dimension of a model. *The annals of statistics* 1978;**6**(2):461-464.
4. Burnham KP, Anderson DR. Multimodel inference understanding AIC and BIC in model selection. *Sociological methods & research* 2004;**33**(2):261-304.
5. Verbyla AP, Cullis BR, Kenward MG, Welham SJ. The analysis of designed experiments and longitudinal data by using smoothing splines. *Journal of the Royal Statistical Society: Series C (Applied Statistics)* 1999;**48**(3):269-311.
6. Efron B. Bootstrap methods: another look at the jackknife. *Breakthroughs in Statistics* Springer, 1992;569-593.
7. Kreiss J-P, Lahiri S. Bootstrap methods for time series. *Handbook of Statistics: Time Series Analysis: Methods and Applications* 2012;**30**(1).
8. SAS I. SAS 9.1. 3 help and documentation. Cary, NC: SAS Institute Inc., Cary, NC, USA, 2002
