# Supplementary material for: Bayesian Model Averaging with Change Points to Assess the Impact of Vaccination and Public Health Interventions
Source: Epidemiology. 2017 Sep 28;28(6):889–97. doi: 10.1097/EDE.0000000000000719 (PMC5617796; doi:10.1097/EDE.0000000000000719)
Supplement: Supplementary file 2 [file ede-28-889-s002.pdf]

```
############################################
### INSTRUCTIONS
############################################

## In the comments below, cp refers to the change point, w/ is "with", w/o
is "without".

## Data set should have these column names: response, months (in orde),
and off_set.

## The researcher should provide the column numbers for the variables that
will be included as predictors in the data set.

## The researcher can provide where to look for the change points: first
and last.

############################################

######################
#### MAIN FUNCTION
######################

############################################


## p_ncp = total prior prob for models w/o cp
## p_cp = total prior prob for models w/ cp
## input_column has the column numbers of the predictors chosen by the
researcher
## first and last are the first and last months to look for a change point,
respectively
## cp_loc is the month of vaccine introduction or any other introduction
of the intervention of interest

model_fit = function(input_column, dataset, first, last, cp_loc)
{

######################
## Create models:
######################

     if(sum(input_column[1:length(input_column)] ==  0)>= 1 )
          n_var = 0
     else
          n_var = length(input_column)


## Add 2 more variables:cp as an intercept and cp as slope, cp ones will
be the last two variables.

     n_var = n_var + 2
```

```
      var.mat <- matrix(NA, 2^(n_var*3), n_var)
      for (i in 1:n_var)
            var.mat[, i] <- sample(c(0, 1), 2^(n_var*3), replace = T)

      var.mat <- unique(var.mat)


## Models w/ cp as intercept and cp as slope are excluded. A model can either
have cp as intercept or cp as slope.

      cancel.out = rep(0, dim(var.mat)[1])

      for(i in 1:dim(var.mat)[1])
      {
            if(var.mat[i, n_var] ==   1 & var.mat[i, (n_var - 1)] ==   1
)
            cancel.out[i] = i
      }
      var.mat = var.mat[cancel.out ==  0, ]


      model.matrix <- matrix(NA, length(var.mat[, 1]), length(var.mat[1,
]))

      for (i in 1:length(var.mat[, 1])) for (j in 1:length(var.mat[1, ]))
            model.matrix[i, j] = ifelse(var.mat[i, j] == 1, paste(' + vart',
'[, ', j, ']', sep = ''), '')


## Models w/o any seasonality
      models = c()

      model.head1 <- "as.integer(response) ~ 1+offset(log(off_set))"
      for (i in 1:length(var.mat[, 1]))
            models[i] <- paste(c(model.head1, model.matrix[i, 1:n_var]),
collapse = "")

## Models w/ 12-month seasonality

      model.head2 <- "as.integer(response) ~
1+offset(log(off_set))+sin12+cos12"
      for (i in 1:length(var.mat[, 1]) )
            models[(i + length(var.mat[, 1]))] <- paste(c(model.head2,
model.matrix[i, 1:n_var]), collapse = "")

## Models w/ 6-month seasonality

model.head3 <- "as.integer(response) ~ 1+offset(log(off_set))+sin6+cos6"

      for (i in 1:length(var.mat[, 1]) )
            models[(i + 2 * length(var.mat[, 1]))] <- paste(c(model.head3,
```

```
model.matrix[i, 1:n_var]), collapse = "")

## Models with both 12-month and 6-month seasonality

	model.head4 <- "as.integer(response) ~ 
1+offset(log(off_set))+sin12+cos12+sin6+cos6"

	for (i in 1:length(var.mat[, 1]) )
		models[(i + 3 * length(var.mat[, 1]))] <- paste(c(model.head4, 
model.matrix[i, 1:n_var]), collapse = "")


##################
### MODEL FIT: We are fitting three types of change point models: no change 
models, change in mean (aka change in intercept) and change in slope (aka 
change in slope)
##################

	n_var = n_var - 2

	off_set = dataset$off_set

	## Models w/ cp: cp_int -> changepoint as intercept, cp_slp-> 
changepoint as slope

	cp_int = seq(1, length(var.mat[, 1]))[var.mat[, (n_var + 1)] ==   1]
	cp_int_models = c(cp_int,  cp_int + length(var.mat[, 1]) , cp_int + 
2*length(var.mat[, 1]), cp_int + 3*length(var.mat[, 1]))


	cp_slp = seq(1, length(var.mat[, 1]))[var.mat[, (n_var + 2)] ==   1]
	cp_slp_models = c(cp_slp, cp_slp + length(var.mat[, 1]) , cp_slp + 
2*length(var.mat[, 1]), cp_slp + 3*length(var.mat[, 1]))

	cp_models = c(cp_int_models, cp_slp_models)

## Models w/o change point

	n_cp_models = seq(1, length(models))[-c(cp_models)]

## Collect all variables besides seasonality into one place: input_column 
are the column numbers of the predictors in the dataset provided by the 
researcher.

	dataset$vart = matrix(NA, nrow = dim(dataset)[1], ncol = (n_var + 2))
	{
		if(n_var !=  0)
		{
			for(tr in 1:n_var)
				dataset$vart[, tr] = dataset[, input_column[tr]]
			colnames(dataset$vart) = 
c(names(dataset)[input_column], "change point as an intercept", "change
```

```
point as a slope")
          }
          else
          {
               colnames(dataset$vart) = c("change point as an
intercept", "change point as a slope")
          }
     }

# Response

     response = dataset$response

### We will need this while distributing the beta estimates to the right
column in "betas" matrix defined below.

     names.to.check <- c("(Intercept)","sin6","cos6","sin12","cos12")
     for (i in 1:(n_var + 2))
          names.to.check[5 + i] = paste('vart', '[, ', i, ']', sep = '')


### Seasonality terms

     dataset$sin12 = sin(2 * 3.14159 * dataset$months/12)
     dataset$cos12 = cos(2 * 3.14159 * dataset$months/12)
     dataset$sin6 = sin(2 * 3.14159 * dataset$months/6)
     dataset$cos6 = cos(2 * 3.14159 * dataset$months/6)


### bics-> collects BIC values, betas -> collects regression coefficient
estimates, sigma.sq -> collects estimated standard errors of the regression
coefficients

### models_inorder -> keeps the order of the models fit


     bics = rep(NA, (length(n_cp_models) + (last - first + 1) *
length(cp_models)))
     betas_random = matrix(NA, length(bics), ncol =
length(dataset$months))
     betas = matrix(0, length(bics), (length(names.to.check)))
     sigma.sq = matrix(0, length(bics), (length(names.to.check)))
     models_inorder = rep(NA, length(bics))
     fitted_gam = matrix(0, ncol = length(bics), nrow = dim(dataset)[1])
     fitted_lme = matrix(0, ncol = length(bics), nrow = dim(dataset)[1])
     se_fitted_gam = matrix(0, ncol = length(bics), nrow = dim(dataset)[1]
)

## The same model is fit at different change points, so we need to keep
track of for which change point is fit: useful information for calculating
IRR
```

```
      change_points = rep(NA, length(bics))

## We are keeping which models have cp as intercept and cp as slope, we
will need this while calculating IRR

      cp_slp_m = rep(0, length(bics))
      cp_int_m = rep(0, length(bics))
      assign("dataset", dataset, envir=globalenv())

## Models w/o cp:

      aa = 1
      for(i in n_cp_models)
      {
                result =
suppressWarnings(try(gamm4(as.formula(models[i]),random=~(1|months),
family = "poisson",  data=dataset), silent = TRUE))

           if (class(result)[1] == "try-error" ||
sum(is.na(coef(result$gam))[1:length(coef(result$gam))]==TRUE)>=1)
      {
           bics[aa] = -100
                models_inorder[aa] = models[i]
                change_points[aa] = 0
                aa = aa + 1
           }
           else
           {

## This part assigns each coefficient to the right column in betas matrix
                similars = rep(0, length(names.to.check))
                for(k in 1:length(names.to.check))
                {
                     for(j in 1:length(coef(result$gam)))
                     {
                          if(names.to.check[k] ==
attributes(coef(result$gam))$names[j])
                          similars[k] = j
                     }
                }

                betas[aa, which(similars!=0)] = coef(result$gam)
                sigma.sq[aa, which(similars!=0)] =
diag(vcov(result$gam))
                betas_random[aa, ] = coef(result$mer)$months[,1]
                bics[aa] = BIC(result$mer)
                models_inorder[aa] = models[i]
                change_points[aa]  = 0
                fitted_gam[,aa] = fitted(result$gam)
                se_fitted_gam[, aa] = predict(result$gam, se.fit =
TRUE)$se.fit
                fitted_lme[, aa] = fitted(result$mer)
```

```
                  aa = aa + 1
            }
      }


## Models w/ cp:

      for(kk in first:last)
      {
            dataset$vart[, (n_var + 1)] = (dataset$months>=
dataset$months[kk]) * 1
            dataset$vart[, (n_var + 2)] = (dataset$months>=
dataset$months[kk]) * (dataset$months - dataset$months[kk])

            assign("dataset", dataset, envir=globalenv())

## Intercept models

            for(i in cp_int_models)
                  {
                        result =
suppressWarnings(try(gamm4(as.formula(models[i]),random=~(1|months),
family = "poisson",  data=dataset), silent = TRUE))

      if (class(result)[1] == "try-error" ||
sum(is.na(coef(result$gam))[1:length(coef(result$gam))]==TRUE)>=1 )
            {
                  bics[aa] = -100
                        models_inorder[aa] = models[i]
                        change_points[aa] = kk
                        cp_int_m[aa] = 1
                        aa = aa + 1

                  }
                  else
                  {
                        similars = rep(0, length(names.to.check))
                        for(k in 1:length(names.to.check))
                        {
                              for(j in 1:length(coef(result$gam)))
                              {
                                    if(names.to.check[k] ==
attributes(coef(result$gam))$names[j])
                                    similars[k] = j
                              }
                        }

                        betas[aa, which(similars!=0)] = coef(result$gam)
                        betas_random[aa, ] = coef(result$mer)$months[,1]

                        sigma.sq[aa, which(similars!=0)] =
diag(vcov(result$gam))
```

```
                        bics[aa] = BIC(result$mer)
                        models_inorder[aa] = models[i]
                        change_points[aa] = kk
                        cp_int_m[aa] = 1
                        fitted_gam[, aa] = fitted(result$gam)
                        se_fitted_gam[, aa] = predict(result$gam, se.fit =
TRUE)$se.fit
                        fitted_lme[, aa] = fitted(result$mer)
                        aa = aa + 1
                  }
            }


## Slope models

      for(i in cp_slp_models)
      {
                  result =
suppressWarnings(try(gamm4(as.formula(models[i]),random=~(1|months),
family = "poisson",  data=dataset), silent = TRUE))

                  if (class(result)[1] == "try-error" ||
sum(is.na(coef(result$gam))[1:length(coef(result$gam))]==TRUE)>=1)
            {
                  bics[aa] = -100
                        models_inorder[aa] = models[i]
                        cp_slp_m[aa] = 1
                        change_points[aa] = kk
                        aa = aa + 1
                  }
                  else
                  {
                        similars = rep(0, length(names.to.check))
                        for(k in 1:length(names.to.check))
                        {
                              for(j in 1:length(coef(result$gam)))
                              {
                                    if(names.to.check[k] ==
attributes(coef(result$gam))$names[j])
                                    similars[k] = j
                              }
                        }
                        betas[aa, which(similars!=0)] = coef(result$gam)
                        betas_random[aa, ] = coef(result$mer)$months[,1]

                        sigma.sq[aa, which(similars!=0)] =
diag(vcov(result$gam))
                        bics[aa] = BIC(result$mer)
                        models_inorder[aa] = models[i]
                        change_points[aa] = kk
                        cp_slp_m[aa] = 1
                        fitted_gam[, aa] = fitted(result$gam)
```

```
                    se_fitted_gam[, aa] = predict(result$gam, se.fit =
TRUE)$se.fit
                    fitted_lme[, aa] = fitted(result$mer)
                    aa = aa + 1

               }
          }
     }

     if(sum(bics==-100) == length(bics)) cat("Did you forget to load
package GAMM4?")


########################
### Calculate the posteriors
########################

     p_ncp = 0.5
     p_cp = 0.5


     l1 = length(models_inorder[bics!= -100])
     l2 = length(n_cp_models)

     l3 = length(models_inorder)

     l4 = length(n_cp_models[bics[1:length(n_cp_models)]!=-100])
     priors = rep(0, l3)
     priors[1:l2] = p_ncp/l4
     priors[(l2 + 1): length(priors)] = p_cp/(l1-l4)

## Model fits that converge will have posteriors, rest won't.

     index = seq(1:length(bics))[bics!= -100]

     delta_bics = rep(0, l3)
     delta_bics = (bics- min((bics[index])))

     posteriors = rep(0, length(bics))
     num_posterior = rep(0, length(bics))
     for(i in index)
          num_posterior[i] =  exp(-0.5 * delta_bics[i]) * priors[i]


     posteriors[index] = num_posterior[index]/sum(num_posterior[index])

## Sum of posterior probabilities for each time point:

     post_one = rep(0, last)


     for(i in first:last)
```

```
            post_one[i] = sum(posteriors[change_points == i])

## Sum of posterior probabilities for change point models

            posterior_cp = 1- round(sum(posteriors[change_points ==  0 & 
bics!= -100]), digits = 5)

#####################
### COUNTERFACTUALS:
#####################

      sin12 = sin(2 * 3.14159 * dataset$months/12)
      cos12 = cos(2 * 3.14159 * dataset$months/12)
      sin6 = sin(2 * 3.14159 * dataset$months/6)
      cos6 = cos(2 * 3.14159 * dataset$months/6)


n = dim(dataset)[1]

### IRR value will be stored in this vector:

      IRR_fitted = rep(0, n)

## Matrix to store counterfactuals, n is the number of months, each column 
will correspond to a different model.

      fitted_gam_ncp = matrix(NA, nrow = dim(fitted_gam)[1], ncol = 
dim(fitted_gam)[2])


## Only focus on models that converged:

      index = seq(1, length(bics))[bics!=-100]

### Matrix for covariates, each model has a different value for change 
points, so we'll fill in the last two columns of X_1 separately for each 
model

      X_1 = cbind(rep(1, n), sin12, cos12, sin6, cos6, dataset[, 
input_column])

cp_loc = cp_loc

      for(j in index)
      {

## For models with no change point we assigned counterfactual to be equal 
to the ones obtained from the model

            if(change_points[j]==0)

                  fitted_gam_ncp[, j] = fitted_lme[, j]
```

```
            else
            {

                  cp1 = (dataset$months>= change_points[j]) * 1  ## change
in mean
                  cp2 = (dataset$months>= change_points[j]) *
(dataset$months - change_points[j])  ## change in slope


                  X_11= cbind(X_1,
cp1*(sum(posteriors[models_inorder==models_inorder[j] &
change_points<=cp_loc])/sum(posteriors[models_inorder==models_inorder[
j]])), cp2*(sum(posteriors[models_inorder==models_inorder[j] &
change_points<=cp_loc])/sum(posteriors[models_inorder==models_inorder[
j]])))


                  fitted_gam_ncp[, j] = exp(betas[j, ] %*% t(X_11) +
betas_random[j,])*(dataset$off_set)
            }
      }

## Model_averaged predictors: weighted_fitted
## Weighted counterfactuals: weighted_fitted_ncp

      weighted_fitted = rep(0, dim(dataset)[1])
      weighted_fitted_ncp = rep(0, dim(dataset)[1])
      weighted_fitted_se = rep(0, dim(dataset)[1])

      for(i in 1:n)
      {
            weighted_fitted_ncp[i] =
(fitted_gam_ncp[i,index])%*%posteriors[index]
            weighted_fitted[i] =
(fitted_lme[i,index])%*%posteriors[index]
            weighted_fitted_se[i] =
(se_fitted_gam[i,index])%*%posteriors[index]
      }


### IRR fitted vector has the IRR values, and IRR_CI_1 and IRR_CI_2 have
the components of our naive confidence interval estimate (Please see
Supplementrary methods for details and comparison of this confidence
interval to bootstrap confdence intervals):

IRR_fitted = weighted_fitted/weighted_fitted_ncp
IRR_CI_1 =
exp(log(weighted_fitted)+1.96*weighted_fitted_se)/weighted_fitted_ncp
IRR_CI_2 =
exp(log(weighted_fitted)-1.96*weighted_fitted_se)/weighted_fitted_ncp
```

```
list(bics = bics, betas = betas, sigma.sq = sigma.sq, models_inorder =
models_inorder, change_points = change_points, n_cp_models = n_cp_models,
cp_int_m = cp_int_m, cp_slp_m = cp_slp_m, posteriors = posteriors,
posterior_ncp = 1-posterior_cp, fitted_gam = fitted_gam, fitted_lme =
fitted_lme, se_fitted_gam = se_fitted_gam, response=  response,
betas_random = betas_random, IRR_fitted  = IRR_fitted, IRR_CI_1 =
IRR_CI_1, IRR_CI_2 = IRR_CI_2, weighted_fitted = weighted_fitted,
weighted_fitted_ncp = weighted_fitted_ncp)

}




######################
### CODE TO RUN:
######################

##packages required
require(gamm4)

## Read the data:

dataset = read.csv('location_of_data')

# Define, input column, first, last, and cp_loc and then run the function
(The following numbers are specific to the dataset provided):

input_column = 0
first = 3
last = 129
cp_loc = 50
result = model_fit(input_column, dataset, first, last, cp_loc)

##Fitted values versus observed values:

dev.new(width = 9.5, height = 5.7)
plot(dataset$months, result$weighted_fitted, type="l", xlab = "months",
ylab = "", lwd = 2)
lines(dataset$months, dataset$response, col="coral", lwd = 2)
lines(dataset$months, result$weighted_fitted_ncp, col="cornflowerblue",
lwd = 2)
legend("topright", col = c("black", "coral", "cornflowerblue"),
c("Observed values", "Fitted values", "Counterfactual predictions"), lty
= c(1,1, 1), lwd = 2)

### Posterior probability of change point models:

print(paste("Probability of any change in the time series is", 1-
result$posterior_ncp))

###IRR with our naive confidence interval estimate:
```

```
dev.new(width = 8.5, height = 4.7)
plot(dataset$months, result$IRR_fitted, type="l", ylim = c(0.6, 1.1), xlab
= "months", ylab = "IRR")
lines(dataset$months, result$IRR_CI_1, lty = 2)
lines(dataset$months, result$IRR_CI_2, lty = 2)
```
