# Supplementary material for: Bayesian Model Averaging with Change Points to Assess the Impact of Vaccination and Public Health Interventions
Source: Epidemiology. 2017 Sep 28;28(6):889–97. doi: 10.1097/EDE.0000000000000719 (PMC5617796; doi:10.1097/EDE.0000000000000719)
Supplement: Supplementary file 3 [file ede-28-889-s003.pdf]

months,off_set,response
1,39000,6879
2,39000,12735
3,39000,13563
4,39000,11381
5,39000,9212
6,39000,10095
7,39000,15647
8,39000,11661
9,39000,11298
10,39000,11131
11,39000,7928
12,39000,4672
13,39000,6421
14,39000,8748
15,39000,19329
16,39000,12532
17,39000,13518
18,39000,7007
19,39000,10574
20,39000,15197
21,39000,12016
22,39000,9070
23,39000,5703
24,39000,4739
25,39000,5418
26,39000,11879
27,39000,16625
28,39000,14742
29,39000,10419
30,39000,9351
31,39000,10013
32,39000,13954
33,39000,15976
34,39000,11298
35,39000,5145
36,39000,4162
37,39000,5562
38,39000,10282
39,39000,11969
40,39000,11641
41,39000,10421
42,39000,9588
43,39000,12778
44,39000,13540
45,39000,7874
46,39000,7615
47,39000,6771
48,39000,4572

49,39000,5931
50,39000,12528
51,39000,17566
52,39000,11114
53,39000,8894
54,39000,8684
55,39000,11299
56,39000,9721
57,39000,14454
58,39000,8565
59,39000,6183
60,39000,4100
61,39000,5497
62,39000,8700
63,39000,17308
64,39000,13194
65,39000,10110
66,39000,6370
67,39000,8778
68,39000,9349
69,39000,11229
70,39000,5867
71,39000,3268
72,39000,3632
73,39000,6221
74,39000,10269
75,39000,11464
76,39000,9941
77,39000,7614
78,39000,9050
79,39000,10656
80,39000,9374
81,39000,9542
82,39000,9100
83,39000,4601
84,39000,3032
85,39000,4136
86,39000,8185
87,39000,8120
88,39000,12028
89,39000,5496
90,39000,6568
91,39000,6603
92,39000,8442
93,39000,10215
94,39000,5422
95,39000,4042
96,39000,3313
97,39000,3222

98,39000,4839
99,39000,7549
100,39000,12769
101,39000,6073
102,39000,5609
103,39000,7284
104,39000,10623
105,39000,6922
106,39000,4708
107,39000,3928
108,39000,2408
109,39000,3764
110,39000,5929
111,39000,7387
112,39000,7322
113,39000,6067
114,39000,5254
115,39000,6117
116,39000,6901
117,39000,6322
118,39000,3880
119,39000,2947
120,39000,1649
121,39000,3578
122,39000,6166
123,39000,6336
124,39000,6177
125,39000,5036
126,39000,5296
127,39000,5067
128,39000,6035
129,39000,6357
130,39000,4395
131,39000,2790
132,39000,2092
